# m6A‐Driven Pexophagy Triggers Placental Ferroptosis to Impair Fetal Growth Upon Environmental Stress

**DOI:** 10.1002/advs.77068

**Published:** 2026-08-12

**Authors:** Xin‐Xin Zhang, Ye‐Xin Luo, Cheng‐Fang Sun, Rui Pan, Yu‐Xi Wu, Zhi Yuan, Xin‐Run Wang, Xu‐Dong Zhang, Lan‐Lan Wu, Xiang‐Yu Yao, Zhan‐Dong Song, Wei Chang, Tian Wei, Pan‐Yuan Cai, Tian‐Ci Zhang, Yong‐Wei Xiong, De‐Xiang Xu, Hua Wang, Hua‐Long Zhu

**Affiliations:** ^1^ Department of Toxicology School of Public Health Anhui Medical University Hefei China; ^2^ Key Laboratory of Environmental Toxicology of Anhui Higher Education Institutes Hefei China; ^3^ The Fourth Affiliated Hospital of Anhui Medical University Anhui Medical University Hefei China; ^4^ Department of Rehabilitation Medicine, and the Second Affiliated Hospital of Anhui Medical University Hefei China

**Keywords:** ferroptosis, FGR, m6A, pexophagy, placenta

## Abstract

The role and underlying mechanisms of placental ferroptosis in fetal growth restriction (FGR) induced by environmental stress remain poorly understood. Our population‐based study showed elevated ferroptosis levels in all‐cause FGR placentae. Environmental stressor cadmium (Cd) was used to generate an FGR mouse model, which exhibited elevated placental ferroptosis. Ferroptosis inhibitor ferrostatin‐1 reversed environmental Cd‐induced FGR. Targeted oxidized lipidomics identified the peroxisome as a target organelle for prenatal Cd‐induced placental ferroptosis. Furthermore, Cd induced excessive activation of PEX5‐dependent pexophagy in placentae. By establishing a placental *Pex5*‐knockdown mouse, pexophagy was confirmed to drive environmental Cd‐induced placental ferroptosis. Mechanistically, pexophagy drives the degradation of the H_2_O_2_‐scavenging enzyme and the fatty‐acid β‐oxidation enzymes, thereby causing lipid peroxidation and placental ferroptosis. Notably, environmental Cd upregulated PEX2, an E3 ligase mediating PEX5 monoubiquitination, thereby driving pexophagy and placental ferroptosis. Furthermore, METTL14‐mediated m6A modification enhanced the stability of placental *PEX2* mRNA in an ELAVL1‐dependent manner under environmental Cd. SAH, a METTL14 inhibitor, alleviated Cd‐induced placental pexophagy, ferroptosis, and FGR. High‐temperature also decreased GPX4 and increased PEX2, PEX5, and METTL14 in placentae. Overall, our findings uncover a novel m6A‐PEX2‐PEX5 axis driving pexophagy‐dependent placental ferroptosis, offering placental pexophagy as a therapeutic target for FGR and fetal‐origin adult diseases.

## Graphical abstract

Prenatal environmental stress exposure promotes m6A modification to drive PEX2‐dependent pexophagy, thereby causing placental ferroptosis and FGR.

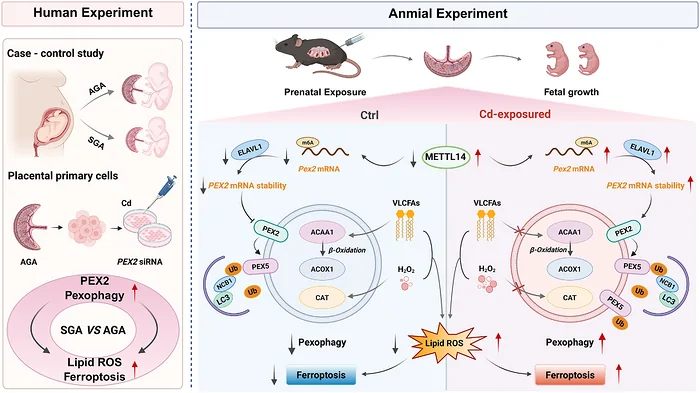

## 1 Introduction

Fetal growth restriction (FGR) is a critical cause of high newborn mortality and increased occurrence of chronic diseases in adulthood, with an incidence rate as high as 10% [1, 2, 3, 4]. Studies have shown that environmental stress can induce FGR by triggering placental programmed cell death, such as apoptosis, autophagy‐dependent cell death, and pyroptosis [5, 6, 7]. Ferroptosis, a novel form of programmed cell death driven by iron‐dependent lipid peroxidation [8]. Under physiological conditions, ferroptosis plays a crucial regulatory role throughout gestation, affecting embryonic development, implantation, and the maintenance of pregnancy [9]. However, under pathological conditions, excessive ferroptosis has been documented in preeclampsia, unexplained miscarriage, and gestational diabetes mellitus, participating in placental dysfunction and the pathogenesis of adverse pregnancy outcomes [10, 11, 12]. Environmental stress exposure has also been demonstrated to trigger ferroptosis in non‐pregnant tissues, including the testes and kidneys, according to prior research [13, 14]. Research has shown that exposure to environmental stress can induce lipid deposition and oxidative stress in placental cells [15, 16]. However, the function and underlying mechanisms of placental ferroptosis in FGR caused by environmental stress remain unclear. Therefore, we hypothesize that environmental stress may induce FGR by aberrantly activating ferroptosis pathways in placental cells.

Pexophagy is a selective autophagy process that targets peroxisomes for degradation by lysosomes [17]. PEX5 monoubiquitination can promote its translocation to peroxisomes, thereby activating pexophagy [18]. Physiologically, pexophagy helps sustain peroxisomal protein homeostasis, thus supporting cellular function and viability [19]. However, under pathological conditions, overactive pexophagy triggers peroxisome protein depletion, which in turn causes cell dysfunction and death [20, 21]. Previous studies have primarily focused on the role of dysregulated pexophagy in the pathogenesis of non‐gestational pathologies, including renal impairment and neurodegenerative processes [22, 23, 24]. However, no studies have hitherto focused on the function of pexophagy in compromising the pregnancy tissue placenta. Peroxisomes not only facilitate the scavenging of hydrogen peroxide (H_2_O_2_), but also serve as the only organelle responsible for degrading very‐long‐chain fatty acids (VLCFAs) [25, 26]. Current evidence suggests that peroxisome dysfunction can lead to the accumulation of lipid peroxides [27]. Previous studies have shown that environmental stress can induce excessive pexophagy, thereby leading to peroxisomal dysfunction in mouse liver and human retinal pigment epithelium [28, 29]. An in vitro study confirmed that viral infection could trigger ferroptosis via excessive pexophagy in A549 cells [30]. Building upon the above findings, this study hypothesized that prenatal maternal environmental stress may induce placental ferroptosis by activating pexophagy.

N6‐methyladenosine (m6A) methylation represents the predominant and most plentiful RNA modification found in eukaryotic cells [31]. m6A methylation is mediated by “writers” (METTL14), eliminated by “erasers” (FTO and ALKBH5), and recognized by “readers” (ELAVL1) to modulate a range of cellular activities, such as proliferation, autophagy, and cell death [32, 33, 34, 35]. In a physiological state, m6A modification contributes to placental development, maintaining normal pregnancy [36]. In pathological conditions, excessive m6A modification impairs placental function, thereby inhibiting fetal growth [37, 38, 39]. Research has shown that environmental stress leads to FGR by enhancing m6A modification in the placentae [40]. Growing evidence suggests that m6A modification promotes non‐selective autophagy activation in the testicle and liver [41, 42, 43]. However, the role of m6A in activating pexophagy, a selective form of autophagy, remains unclear. PEX2, PEX10, and PEX12, three E3 ubiquitin ligases, reportedly catalyze PEX5 monoubiquitination, thereby activating pexophagy [44]. According to a recent report, m6A modification activates placental ER‐phagy by upregulating the expression of the ER‐phagy receptor RTN3L [45]. Thus, we hypothesized that prenatal environmental stress exposure activates placental pexophagy by promoting m6A modification.

An animal model of FGR induced by the prenatal environmental stressor Cd was established in this study. Ferrostatin‐1 (Fer‐1) was applied to explore the function of placental ferroptosis in FGR triggered by prenatal environmental stress. Placental *Pex5*‐specific knockdown mice were created to elucidate the impact of pexophagy activation on placental ferroptosis induced by prenatal environmental stressor Cd and its mechanism. PEX2‐deficient human placental cells were generated to elucidate the mechanism through which prenatal environmental stressor Cd activates placental pexophagy. In vitro *METTL14/ELAVL1* knockdown and in vivo S‐adenosylhomocysteine (SAH) supplementation were conducted to examine the impact of m6A modification on prenatal environmental Cd‐activated placental PEX2‐dependent pexophagy. This study will offer a scientific foundation for understanding the mechanisms by which environmental toxins induce FGR and related fetal‐origin diseases.

## 2 Results

### 2.1 Placental Ferroptosis Links Prenatal Environmental Stress to FGR

A population study was initially designed to evaluate the association between placental ferroptosis and FGR. Out of 183 potential samples with available maternal‐infant data and placental tissue, 114 were successfully enrolled following a rigorous screening process (Figure S1A). Placental malondialdehyde (MDA), a marker of ferroptosis, was negatively correlated with both birth weight and placental weight (Figure 1A–C and Figure S1B–E). MDA level was also higher in the small‐for‐gestational‐age (SGA) placentae than that in the appropriate‐for‐gestational‐age (AGA) placentae (Figure S1F). Furthermore, higher placental MDA levels were associated with lower birth weight in both male and female infants, with no sex difference in MDA levels (Figure S1G–I). Transmission electron microscopy (TEM) results showed that SGA placentae exhibited ferroptosis‐related mitochondrial phenotypes, including cristolysis and mitochondrial atrophy (Figure 1D). Further results showed that 4‐HNE, a terminal product of lipid peroxidation, also accumulated in SGA placentae. Compared with AGA placentae, SGA placentae exhibited decreased expression of GPX4, which is a key inhibitor of ferroptosis. In addition, the expression of the key ferroptosis driver ACSL4 was upregulated in the SGA placentae (Figure 1E and Figure S1J,K).

**FIGURE 1:**
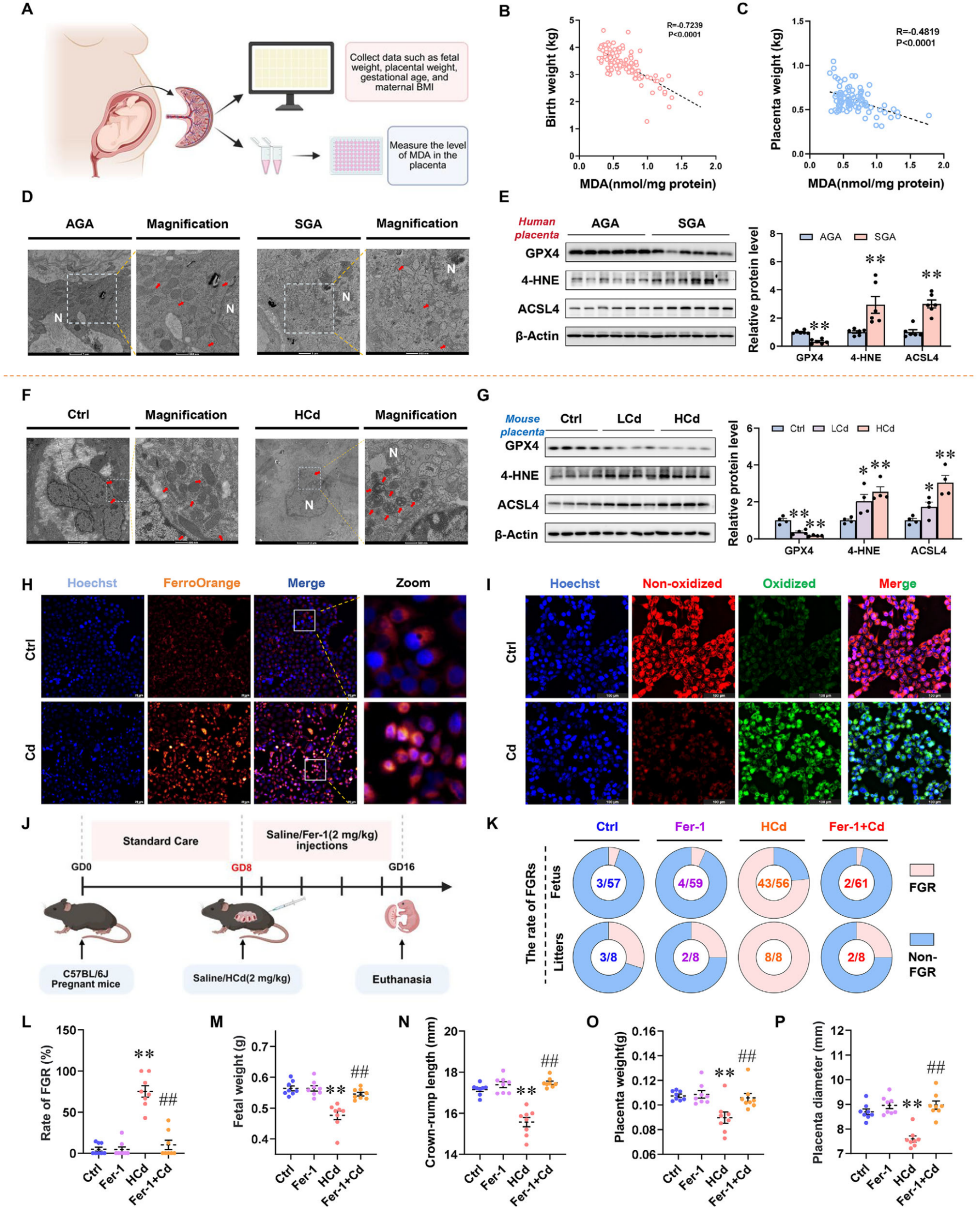
Placental ferroptosis links prenatal environmental stress to FGR. (A–E) A total of 183 maternal‐infant pairs were recruited from the Fourth Affiliated Hospital of Anhui Medical University, of which 114 placental samples were finally included in the cross‐sectional analysis. Informed consent was obtained from all participants. (A) Human experiment diagram. (B) Linear relationship between birth weight and MDA. (C) Linear relationship between placental weight and MDA. (D) TEM images of human placental cells. (E) Levels of GPX4, 4‐HNE, and ACSL4 in human placentae. (F, G) Pregnant mice received intraperitoneal administration of low or high doses of CdCl_2_ on GD 8. Placentae were obtained on GD 16 subsequent to euthanasia. (F) TEM images of mouse placental cells. (G) Levels of GPX4, 4‐HNE, and ACSL4 proteins in mouse placentae. (H, I) The human JEG‐3 cells received CdCl_2_ (20 µm) for 12 h. FerroOrange and C11‐BODIPY staining of cells. (J–P) On GD 8, pregnant mice from the HCd and Fer‐1+HCd groups received intraperitoneal administration of CdCl_2_ at 2 mg/kg. Pregnant mice from the Fer‐1 group and Fer‐1+HCd group received intraperitoneal administration of 2 mg/kg Fer‐1 on GD 8, 9, 11, 13, and 15, with Fer‐1 administered 30 min before the intraperitoneal injection of CdCl_2_. After euthanasia, the placentae were collected on GD 16. (J) Animal experiment diagram of Fer‐1. (K) Number of litters and number of pups with FGR phenotype. (L) Incidence of FGR. (M) Fetal mouse weight. (N) Fetal mouse crown‐rump length. (O) Placental weight. (P) Placental diameter. *n* = 4–8. ^*^ *P* < 0.05, ^**^ *P* < 0.01 vs. AGA or Ctrl; ^##^ *P* < 0.01 vs. HCd.

Subsequently, Cd was employed to investigate how prenatal environmental stress affects placental ferroptosis and fetal growth. Serum Cd levels in the low‐ and high‐Cd groups were 1.34 ± 0.09 and 2.30 ± 0.05 µg/L, respectively (Figure S2A), overlapping with the ranges reported in human studies [46, 47, 48]. Cd exposure resulted in an increased rate of FGR, as well as decreased fetal and placental sizes (Figure S3A–G). TEM analysis indicated that Cd exposure also induced cristolysis and mitochondrial atrophy in mouse placentae (Figure 1F). Correspondingly, Cd exposure downregulated GPX4 expression while upregulating 4‐HNE and ACSL4 expression in mouse placentae (Figure 1G and Figure S3J,L). Accordingly, Cd exposure led to elevated MDA levels and reduced GSH levels in mouse placentae (Figure S3H,I). Moreover, Cd exposure significantly increased 4‐HNE levels in placental CK7‐positive cells, a marker of placental trophoblasts (Figure S2B,C). Primary trophoblast cells were subsequently isolated and purified from mouse placentae in each group, and their intracellular ferrous ion (Fe^2^^+^) levels were measured. The results indicated that Fe^2+^ was elevated in Cd‐exposed placentae (Figure S3K,M). Cd exposure specifically impaired mouse labyrinth zone (LZ), as indicated by a reduced LZ/ junctional zone (JZ) area ratio and decreased expression of the LZ marker GCM1, without affecting the JZ marker TPBPA (Figure S2D–G). Cd exposure also impaired placental syncytialization and invasion, as evidenced by reduced *Syna, Synb*, and *Gcm1* mRNA, along with downregulated total MMP2, active MMP2, and N‐cadherin expression, respectively (Figure S2H–L). Given the high similarity to LZ trophoblasts, human JEG‐3 cells were used for the in vitro experiments. The data showed that Cd exposure reduced GPX4 in JEG‐3 cells in a dose‐ and time‐dependent manner, while increasing 4‐HNE and ACSL4 (Figure S4A–G). Immunofluorescence analysis further confirmed significant accumulation of 4‐HNE within these cells (Figure S4H,I). Furthermore, Cd exposure resulted in increased Fe^2^^+^ and oxidized lipid levels within JEG‐3 cells (Figure 1H,I and Figure S4J,K). In summary, these findings substantiate that Cd exposure precipitates placental ferroptosis, thereby driving the pathogenesis of FGR.

Fer‐1, a ferroptosis inhibitor, primarily works by scavenging free radicals to inhibit lipid peroxidation [49]. Pregnant mice were administered Fer‐1 to examine how placental ferroptosis contributes to FGR caused by Cd exposure (Figure 1J). The findings indicated that Fer‐1 markedly attenuated the increased incidence of FGR induced by Cd exposure (Figure 1K,L). Compared with the HCd group, the Fer‐1+HCd group exhibited higher fetal and placental weights, as well as greater crown‐rump length and placental diameter (Figure 1M–P and Figure S5A). Furthermore, Fer‐1 restored the Cd‐induced reduction in GSH levels and elevation in MDA levels within the placenta (Figure S5B,C). Immunofluorescence data demonstrated that Fer‐1 potently suppressed the Cd‐induced elevation of 4‐HNE in mouse placentae (Figure S5D,E). TEM analysis demonstrated that Fer‐1 reduced ferroptosis‐related mitochondrial phenotypes, in the placenta, such as cristolysis and atrophy, under Cd exposure. Moreover, the number of lipid droplets in the Fer‐1+HCd group was reduced relative to the HCd group (Figure S5F). To determine placental Fe^2^^+^ levels, the mouse placental primary trophoblast cells were isolated from the mouse placentae. The analysis demonstrated that the Fe^2^^+^ level in the Fer‐1+HCd group was markedly reduced relative to the HCd group (Figure S5G,H). Relative to the HCd group, the Fer‐1+HCd group displayed increased GPX4 levels and decreased 4‐HNE and ACSL4 levels in placentae (Figure S5I–L). In summary, our findings suggest that environmental stress causes FGR via triggering placental ferroptosis.

### 2.2 Prenatal Environmental Stress Triggers Placental Pexophagy

Lipid peroxidation has been established as a key mechanism underlying ferroptosis [50]. To investigate how Cd exposure induces placental ferroptosis, targeted oxidized lipidomics of mouse placentae was performed. Targeted oxidized lipidomics revealed significant accumulation of oxidized lipids, specifically very‐long‐chain fatty acids (VLCFAs, C ≥ 22), in response to Cd exposure (Figure 2A,B and Figure S6A,B). Our data indicated that the levels of ABCD2 and PMP70, two peroxisomal markers, were significantly reduced in placentae following Cd exposure. However, PGC1α expression did not differ among groups, indicating that peroxisome biogenesis was not significantly altered. The levels of LC3B‐II and NBR1, two markers of autophagy, were increased in Cd‐exposed placentae (Figure 2C). Ultrastructural analysis via TEM further evidenced a marked increase in the abundance of placental autophagosomes under Cd exposure (Figure 2D). Further data confirmed that Cd exposure activated pexophagy in the placentae, as evidenced by the elevation of PEX5, and increased co‐localization of LC3B and PEX5 (Figure 2C,E and Figure S6C). We further explored the effect of Cd exposure on pexophagy in human placental trophoblasts. As predicted, JEG‐3 cells subjected to Cd exposure exhibited decreased ABCD2 and PMP70, together with a rise in PGC1α levels. Cd exposure also elevated the levels of pexophagy marker LC3B‐II, NBR1, and PEX5 (Figure 2F). According to subsequent CHX chase assays, Cd exposure substantially promoted the degradation rate of ABCD2 in human JEG‐3 cells, which implies diminished protein stability (Figure 2G). Chloroquine (CQ), an inhibitor of lysosomes, was used to treat JEG‐3 cells to further assess the effect of Cd exposure on pexophagy flux. The data indicated that treatment with CQ effectively prevented the loss of ABCD2 caused by Cd exposure, confirming that Cd exposure activates pexophagy flux (Figure 2H). Collectively, the above findings suggest that environmental stress activates pexophagy in the placenta.

**FIGURE 2:**
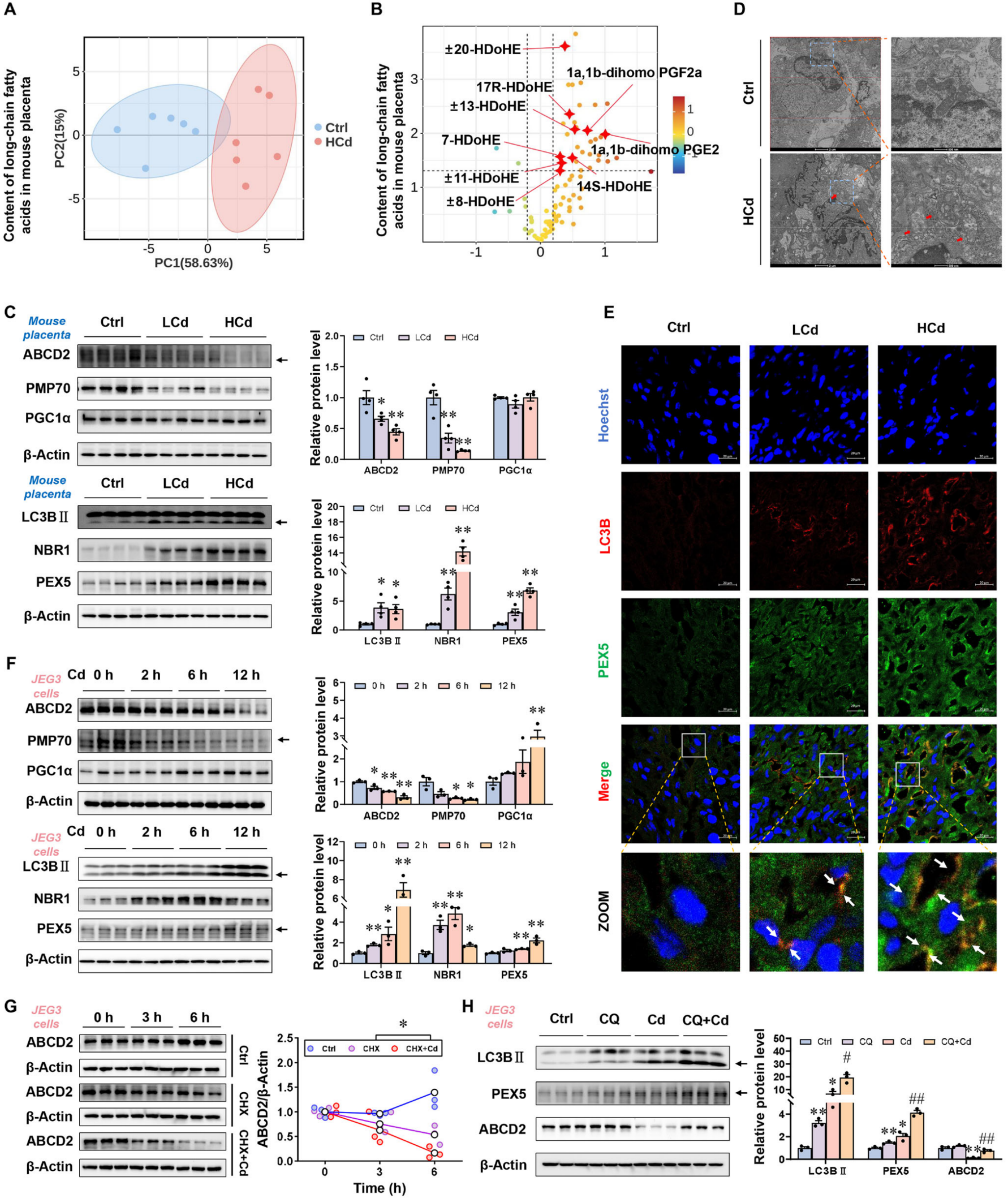
Prenatal environmental stress triggers placental pexophagy. (A–E) Pregnant mice received intraperitoneal administration of low or high doses of CdCl_2_ on GD 8. Placentae were obtained on GD 16 subsequent to euthanasia. (A) Principal component analysis (PCA) plot of placental lipidomics. (B) Volcano plot of placental lipidomics. (C) Levels of ABCD2, PMP70, PGC1α, LC3B‐II, NBR1, and PEX5 in placentae. (D) TEM images of placental cells. (E) Immunofluorescent detection of LC3B and PEX5 in mouse placentae. (F) Levels of ABCD2, PMP70, PGC1α, LC3B‐II, NBR1, and PEX5 in JEG‐3 cells. (G) ABCD2 degradation in JEG‐3 cells following treatment with CHX alone or in combination with Cd. (H) Protein levels of PEX5, ABCD2, and LC3B‐II. The cells received CQ pretreatment prior to CdCl_2_ exposure. *n* = 3–7. ^*^ *P* < 0.05, ^**^ *P* < 0.01 vs. Ctrl or 0 h; *^#^P* < 0.05, ^##^ *P* < 0.01 vs. Cd.

### 2.3 PEX5‐Dependent Pexophagy Drives Placental Ferroptosis Upon Environmental Stress

Monoubiquitinated PEX5 functions as a key signal that marks peroxisomes for pexophagy. To explore how activated pexophagy contributes to placental ferroptosis and FGR induced by Cd exposure, we generated a placental‐specific *Pex5*‐knockdown mouse model (Figure 3A). The results showed that *Pex5* knockdown significantly attenuated the Cd‐induced rise in FGR incidence (Figure 3B and Figure S7A). As expected, *Pex5* knockdown also restored fetal weight and body length under Cd exposure (Figure S7B,C). In the placental *Pex5* knockdown mouse, the decreases in placental weight and diameter induced by Cd exposure were mitigated (Figure S7D,E). Immunofluorescence results revealed that *Pex5* knockdown markedly decreased the deposition of 4‐HNE in Cd‐exposed placentae (Figure 3C,D). TEM results showed that *Pex5* knockdown also reversed the ferroptosis‐related mitochondrial phenotypes evoked by Cd exposure, such as cristolysis and mitochondrial atrophy (Figure 3E). The levels of GPX4 protein and GSH were higher in the *Pex5*‐knockdown placentae with HCd treatment, when compared to HCd‐exposed placentae (Figure 3F,H). The levels of 4‐HNE and ACSL4 protein and the content of MDA were decreased in the *Pex5*‐knockdown placentae under Cd exposure (Figure 3F,G). Furthermore, *Pex5* knockdown reduced the abundance of autophagosomes in placentae exposed to Cd exposure (Figure S7F). Immunofluorescence analysis further confirmed that *Pex5* knockdown inhibited pexophagy induced by Cd exposure, as evidenced by the decreased co‐localization of LC3B and PEX5 (Figure 3I,J). *Pex5* knockdown also reversed Cd‐induced reduction of PMP70 in placentae (Figure 3K). To examine how pexophagy affects Cd‐induced ferroptosis in human placental trophoblast cells, we subsequently established PEX5‐deficient JEG‐3 cells. The results showed that PEX5 deficiency inhibited the decrease of GPX4 in JEG‐3 cells induced by Cd exposure. The levels of 4‐HNE and ACSL4 were decreased in PEX5‐deficient cells under Cd exposure (Figure 3L). Meanwhile, PEX5 deficiency suppressed the accumulation of oxidized lipids in Cd‐exposed cells (Figure S8A). Further data confirmed that PEX5 deficiency blocked pexophagy activation in human JEG‐3 cells under Cd exposure, as evidenced by increased PMP70 levels (Figure 3M). Overall, environmental stress induces placental ferroptosis by activating pexophagy.

**FIGURE 3:**
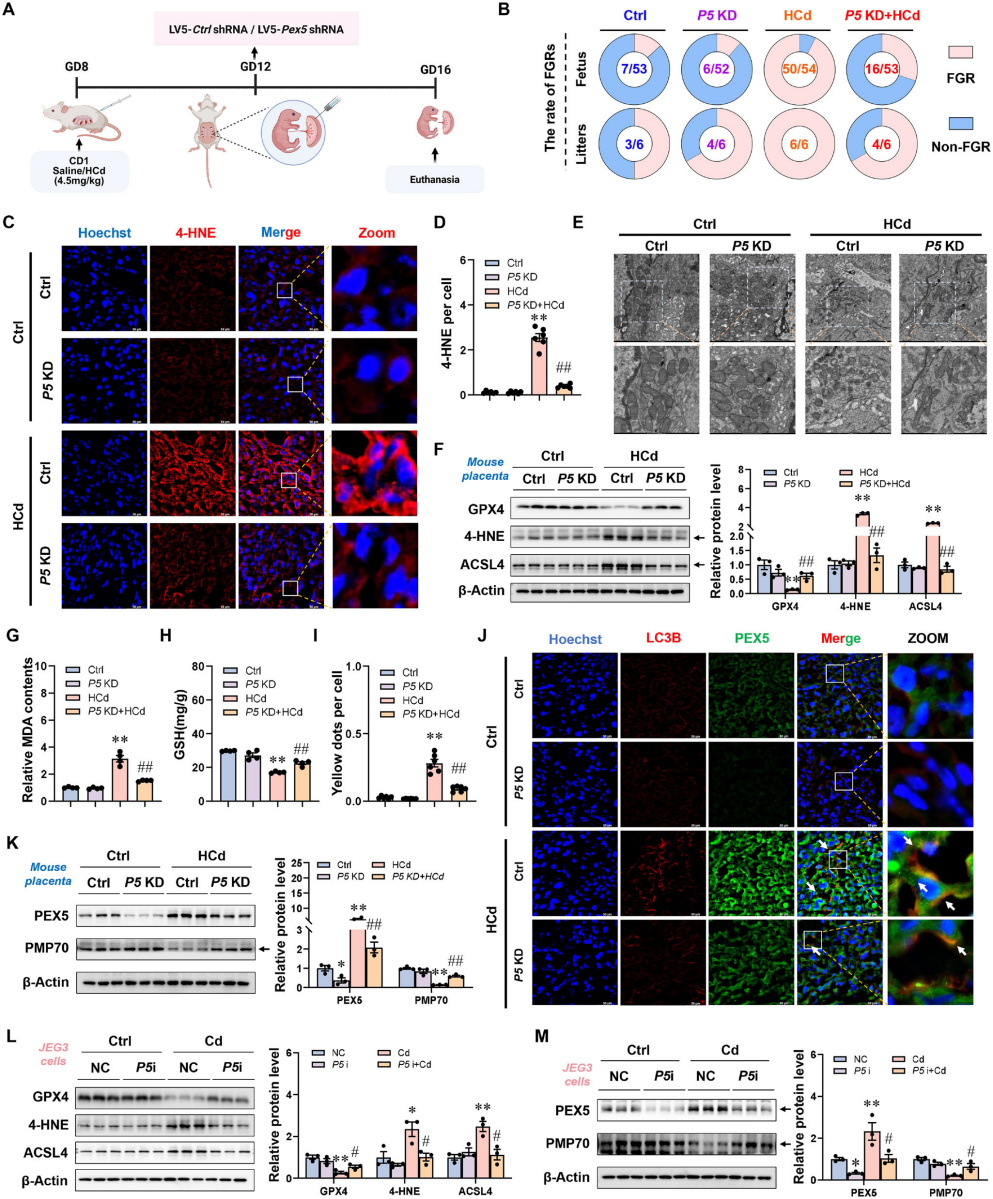
PEX5‐dependent pexophagy drives placental ferroptosis upon environmental stress. (A–K) Intraperitoneally injected high‐concentration CdCl_2_ (2 mg/kg) into pregnant mice of the LV5 *Ctrl* shRNA + Cd group and LV5 *Pex5* shRNA + Cd group on GD 8. All pregnant mice were infected with LV5 *Pex5* shRNA or LV5 *Ctrl* shRNA via placental local injection on GD 12. Euthanized the mice on GD 16, collected placentae. (A) Animal experiment diagram of LV5 *Pex5* shRNA. (B) Number of litters and number of pups with FGR phenotype. (C, D) 4‐HNE immunostaining of placentae. (E) TEM images of placental cells. (F) Levels of GPX4, 4‐HNE, and ACSL4 in placentae. (G) Mouse placental MDA content. (H) Mouse placental GSH content. (I, J) Immunofluorescent detection of LC3B and PEX5 in mouse placentae. (K) Levels of PEX5 and PMP70 in placentae. (L, M) Levels of GPX4, 4‐HNE, ACSL4, PEX5, and PMP70 in JEG‐3 cells. The cells received CdCl_2_ stimulation subsequent to *PEX5* siRNA transfection. *n* = 3–6. ^*^ *P* < 0.05, ^**^ *P* < 0.01 vs. Ctrl or NC; *^#^P* < 0.05, ^##^ *P* < 0.01 vs. HCd or Cd.

### 2.4 Pexophagy Promotes Lipid Peroxidation Accumulation to Drive Placental Ferroptosis Upon Environmental Stress

Peroxisomes eliminate VLCFAs via β‐oxidation and detoxify H_2_O_2_ through CAT, thereby preventing the accumulation of lipid peroxides (Figure 4A). To elucidate the underlying mechanism by which pexophagy mediates Cd‐induced placental ferroptosis, we examined peroxisomal β‐oxidation and H_2_O_2_ detoxification. Cd exposure was found to reduce the expression of ACAA1 and ACOX1 in the placenta, both of which are critical enzymes for peroxisomal β‐oxidation (Figure 4B). Moreover, the level of placental CAT was reduced upon Cd exposure (Figure 4B). Evidence from TEM and immunofluorescence indicated that Cd exposure increases lipid droplet accumulation in the placenta (Figure S9A–C). Besides, placental H_2_O_2_ levels were increased following Cd exposure (Figure S9D). Most importantly, placental‐specific *Pex5* knockdown significantly mitigated the decrease of ACOX1 and CAT induced by Cd exposure (Figure 4C). Meanwhile, the level of H_2_O_2_ was lower in the *Pex5*‐knockdown placentae with HCd treatment, when compared to HCd‐exposed placentae (Figure 4D). TEM and immunofluorescence analysis further confirmed that *Pex5* knockdown significantly inhibited lipid droplet accumulation in placentae exposed to Cd (Figure 4E,F and Figure S9E). We further investigated the effects of Cd‐activated pexophagy on β‐oxidation and ROS metabolism in human placental trophoblast cells. The findings suggested that after CdCl_2_ exposure, cellular levels of ACAA1, ACOX1, and CAT were reduced (Figure S10A). With the DCF probe, Cd exposure was observed to elevate ROS levels in cells (Figure S10B,C). Immunofluorescence results showed that lipid droplet accumulation was enhanced by Cd exposure (Figure S10D,E). Besides, the results showed that Cd exposure promoted the degradation of ACOX1 and CAT proteins in JEG‐3 cells treated with CHX (Figure 4G,H). We next constructed PEX5‐deficient JEG‐3 cells to further investigate the mechanism by which pexophagy mediates Cd‐induced placental ferroptosis. The results showed that, compared with the Cd group, ACOX1 and CAT levels were higher in PEX5‐deficient cells under Cd exposure (Figure 4I). PEX5 deficiency also reversed the Cd‐induced increase in ROS in placentae (Figure 4J). Moreover, PEX5 deficiency reduced the number of lipid droplets in cells exposed to Cd (Figure 4K and Figure S10F). In summary, pexophagy mediates environmental stress‐induced placental ferroptosis by elevating lipid and ROS levels.

**FIGURE 4:**
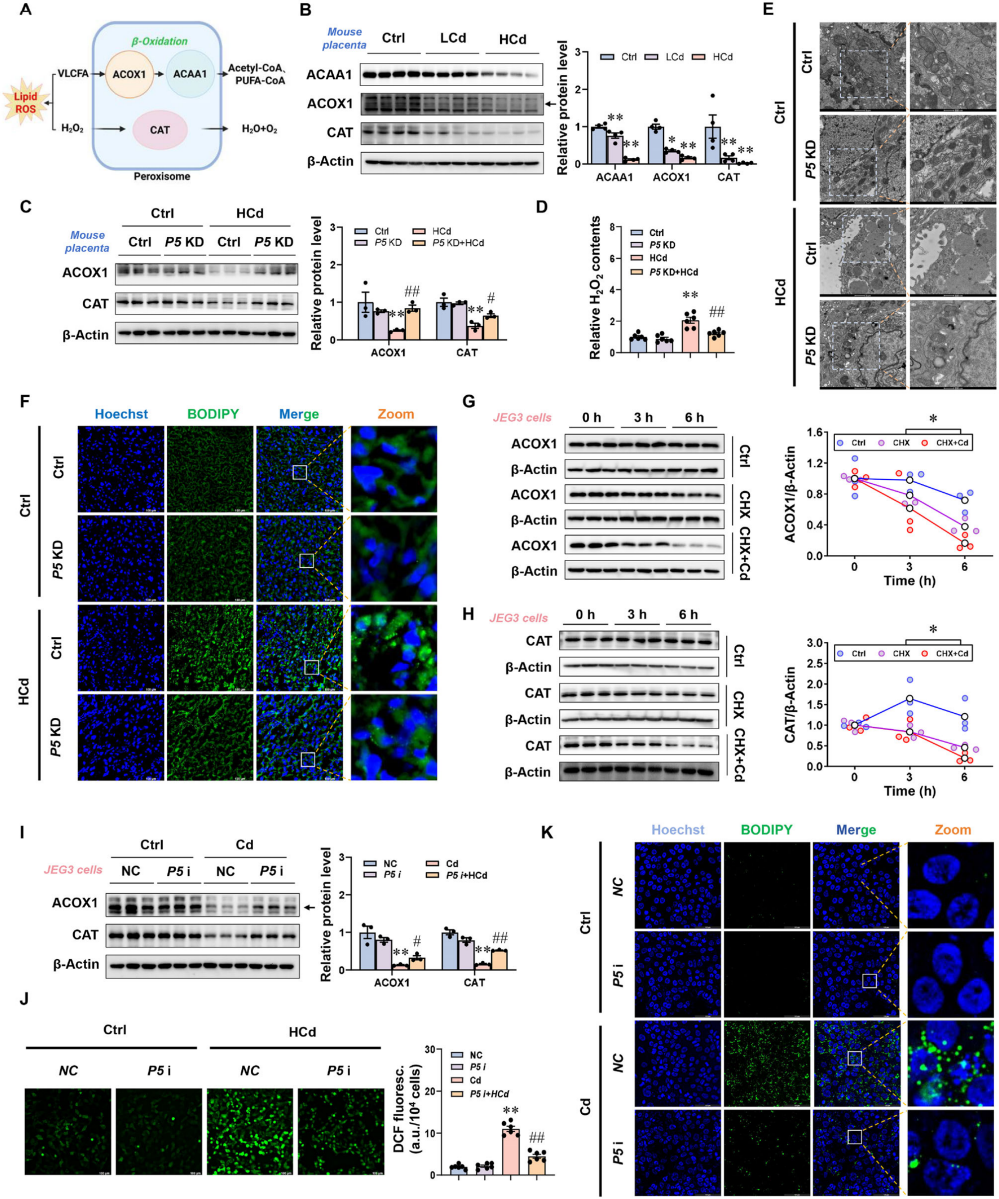
Pexophagy promotes lipid peroxidation accumulation to drive placental ferroptosis upon environmental stress. (A) Schematic diagram of peroxisome function. (B) Levels of ACAA1, ACOX1, and CAT in the placenta. Pregnant mice received intraperitoneal administration of low or high doses of CdCl_2_ on GD 8. Placentae were obtained on GD 16 subsequent to euthanasia. (C–F) Intraperitoneally injected high‐concentration CdCl_2_ (2 mg/kg) into pregnant mice of the LV5 *Ctrl* shRNA + Cd group and LV5 *Pex5* shRNA + Cd group on GD 8. All pregnant mice were infected with LV5 *Pex5* shRNA or LV5 *Ctrl* shRNA via placental local injection on GD 12. The mice were euthanized on GD 16, and the placentae were collected. (C) Levels of ACOX1 and CAT in the placenta. (D) H_2_O_2_ content in the placenta. (E) TEM images of placental cells. (F) BODIPY immunostaining of placentae. (G, H) ACOX1 and CAT degradation in JEG‐3 cells following treatment with CHX alone or in combination with Cd. (I–K) The cells received CdCl_2_ stimulation subsequent to *PEX5* siRNA transfection. (I) Levels of ACOX1 and CAT in JEG‐3 cells. (J) DCFH‐DA immunostaining of JEG‐3 cells. (K) BODIPY immunostaining of JEG‐3 cells. *n* = 3–6. ^*^ *P* < 0.05, ^**^ *P* < 0.01 vs. Ctrl, CHX or NC; *^#^P* < 0.05, ^##^ *P* < 0.01 vs. HCd or Cd.

### 2.5 Prenatal Environmental Stress Elevates PEX2 to Activate Placental Pexophagy

It is well known that PEX2, PEX10, and PEX12 contribute to PEX5 monoubiquitination, thereby activating pexophagy. In contrast, PEX1, PEX6, and PEX26 block pexophagy by deubiquitinating PEX5. The mRNA levels of the above‐mentioned *Pexs* were assessed to uncover the mechanism of pexophagy induced by Cd exposure. According to Figure 5A, Cd exposure markedly upregulated the mRNA expression of *Pex2*, *Pex10*, and *Pex12*. Among these, *Pex2* exhibited the greatest fold increase (3.44), compared to *Pex10* (1.52) and *Pex12* (1.51). Moreover, PEX2 protein levels were also increased in mouse placentae and JEG‐3 cells under Cd exposure (Figure S11A,B). Therefore, PEX2‐deficient JEG‐3 cells were constructed to explore the function of PEX2 in pexophagy triggered by Cd exposure. Besides, PEX2 deficiency reversed the drop in PMP70 caused by Cd exposure in JEG‐3 cells (Figure 5B). The levels of CAT and ACOX1 were also higher in the PEX2‐deficient cells under Cd exposure (Figure 5C,D). We also found that ROS levels were reduced in PEX2‐deficient JEG‐3 cells after exposure to Cd (Figure 5E,G). In addition, PEX2 deficiency significantly suppressed Cd‐evoked lipid droplet accumulation in JEG‐3 cells (Figure 5F,H). Finally, we investigated the effect of PEX2 deficiency on placental ferroptosis upon Cd exposure. The results indicated that PEX2 deficiency could reverse the reduction of GPX4 induced by Cd exposure in JEG‐3 cells (Figure 5I). PEX2 deficiency reduced the levels of 4‐HNE and ACSL4 in Cd‐exposed JEG‐3 cells (Figure 5I). The accumulation of oxidative lipids was significantly suppressed in PEX2‐deficient JEG‐3 cells under Cd exposure (Figure 5J,L). The level of Fe^2+^ was decreased in the PEX2‐deficient JEG‐3 cells under Cd exposure (Figure 5K,M). Overall, environmental stress activates placental pexophagy via upregulating PEX2 expression.

**FIGURE 5:**
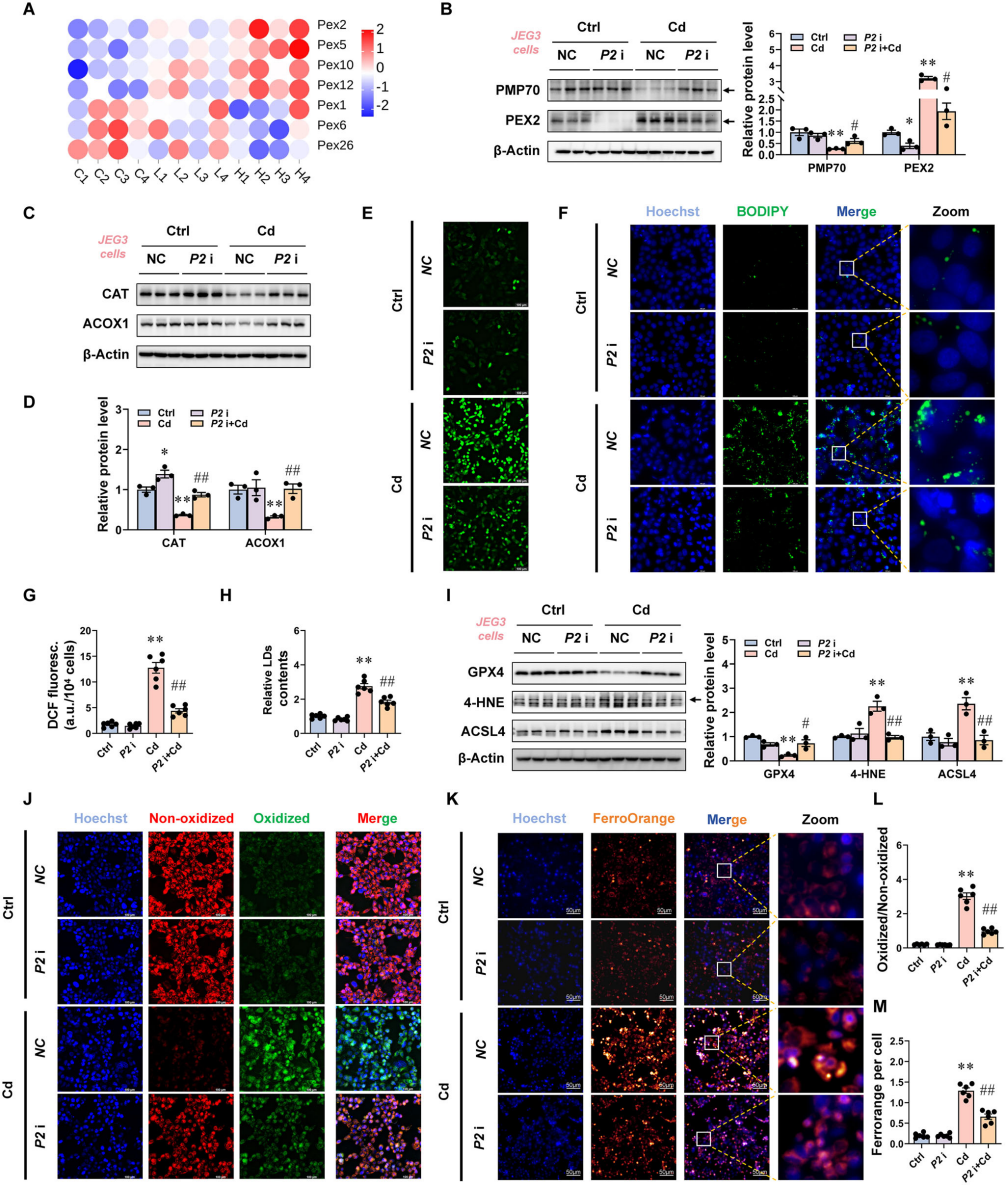
Prenatal environmental stress elevates PEX2 to activate placental pexophagy. (A) mRNA levels of pexophagy receptors in mouse placentae. Pregnant mice received intraperitoneal administration of low or high doses of CdCl_2_ on GD 8. Placentae were obtained on GD 16 subsequent to euthanasia. (B–M) The cells received CdCl_2_ stimulation subsequent to *PEX2* siRNA transfection. (B) Levels of PMP70 and PEX2 in JEG‐3 cells. (C, D) Levels of CAT and ACOX1 in JEG‐3 cells. (E, G) DCFH‐DA immunostaining of JEG‐3 cells. (F, H) BODIPY immunostaining of JEG‐3 cells. (I) Levels of GPX4, 4‐HNE, and ACSL4 in JEG‐3 cells. (J, L) C11‐BODIPY immunostaining of JEG‐3 cells. (K, M) FerroOrange immunostaining of JEG‐3 cells. *n* = 3–6. ^*^ *P* < 0.05, ^**^ *P* < 0.01 vs. Ctrl; *^#^P* < 0.05, ^##^ *P* < 0.01 vs. Cd.

### 2.6 m6A Modification Promotes Placental PEX2 Expression Upon Environmental Stress

To investigate how environmental stressor Cd upregulates PEX2 expression, human JEG‐3 cells were exposed to the transcription inhibitor ActD. The findings demonstrated that exposure to Cd markedly suppressed the degradation of *PEX2* mRNA (Figure 6A). Meanwhile, the placentae showed enhanced total m6A modifications upon exposure to Cd (Figure 6B). According to the SRAMP database, multiple high‐confidence m6A‐methylated sites were identified within *Pex2* mRNA (Figure 6C). MeRIP‐PCR analysis confirmed that prenatal Cd exposure increases the level of m6A‐modified *Pex2* mRNA in placental tissues (Figure 6D and Figure S12A). Based on the RM2Target database, the potential candidates regulating m6A modification in *Pex2* mRNA include m6A writer METTL14, the reader ELAVL1, and the erasers FTO and ALKBH5. Results demonstrated that METTL14 and ELAVL1 protein levels were increased in placentae exposed to Cd (Figure 6E–G). In contrast, the levels of FTO and ALKBH5 remained unchanged after Cd exposure (Figure S12B–D). To further investigate how m6A modification contributes to the upregulation of PEX2 expression under Cd exposure, we constructed METTL14‐deficient JEG‐3 cells. METTL14 deficiency promoted the decay of *PEX2* mRNA in Cd‐exposed JEG‐3 cells (Figure 6H). Immunofluorescence assay results revealed that METTL14 deficiency significantly attenuated the buildup of oxidized lipids in Cd‐exposed cells (Figure 6I,J). The levels of PEX2, 4‐HNE, and ACSL4 were lower in the METTL14‐deficient JEG‐3 cells with Cd treatment relative to the Cd group. Besides, the expression of CAT, ACOX1, PMP70, and GPX4 was upregulated in the METTL14‐deficient JEG‐3 cells under Cd exposure (Figure 6K and Figure S13A,B). We further investigated the role of the m6A reader ELAVL1 in regulating PEX2 expression under Cd exposure. Similarly, ELAVL1 deficiency promoted the decay of *PEX2* mRNA in JEG‐3 cells induced by Cd exposure (Figure 6L). ELAVL1 deficiency markedly attenuated Cd‐induced accumulation of oxidized lipids in JEG‐3 cells (Figure 6M,N). The levels of PEX2, 4‐HNE, and ACSL4 were reduced in ELAVL1‐deficient JEG‐3 cells under Cd exposure. These cells further exhibited higher levels of CAT, ACOX1, PMP70, and GPX4 (Figure 6O and Figure S13C,D). In conclusion, environmental stress drives PEX2‐dependent pexophagy through enhancing m6A modification.

**FIGURE 6:**
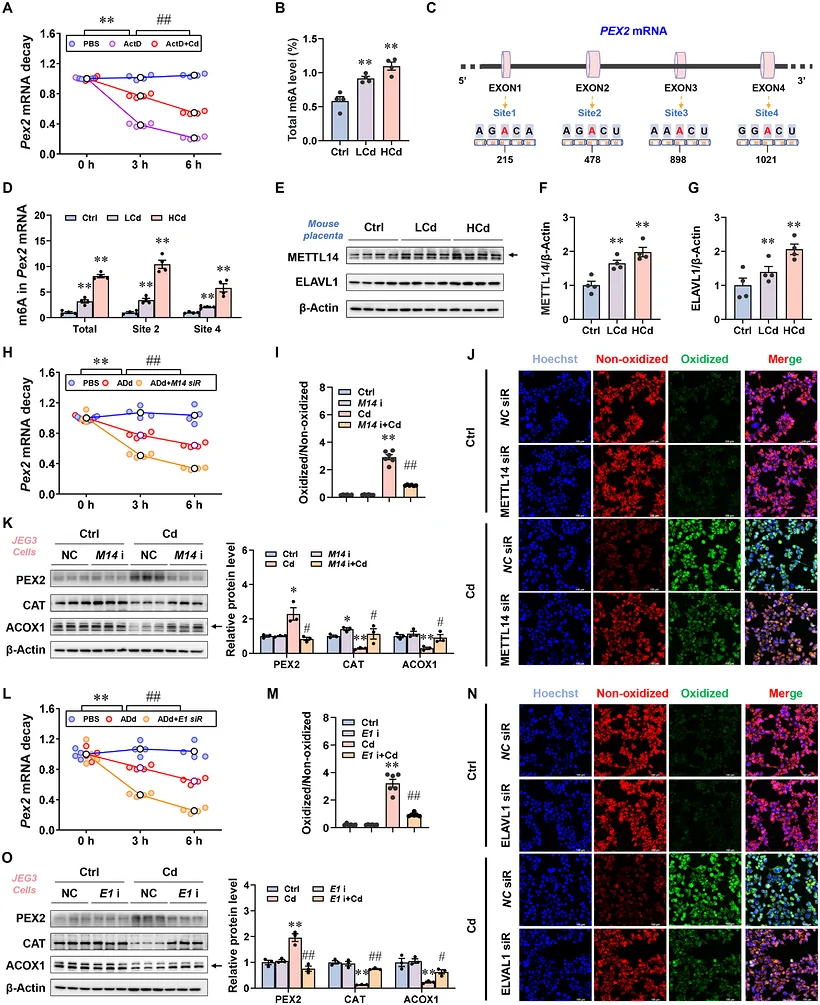
m6A modification promotes placental PEX2 expression upon environmental stress. (A) JEG‐3 cells were pre‐incubated with ActD and subsequently exposed to CdCl_2_ to assess *PEX2* mRNA decay. (B–E) Pregnant mice received intraperitoneal administration of low or high doses of CdCl_2_ on GD 8. Placentae were obtained on GD 16 subsequent to euthanasia. (B) Total m6A content in mouse placental mRNA. (C) m6A methylation site within *PEX2* mRNA. (D) m6A level in *Pex2* mRNA from placentae. (E–G) Levels of METTL14 and ELAVL1 in placentae. (H–K) The cells received CdCl_2_ stimulation subsequent to *METTL14* siRNA transfection. (H) *PEX2* mRNA decay in JEG‐3 cells. Cells were pretreated with ActD and transfected with *METTL14* siRNA before being stimulated with CdCl_2_. (I, J) C11‐BODIPY immunostaining of JEG‐3 cells. (K) Levels of PEX2, CAT, and ACOX1 proteins in JEG‐3 cells. (L–O) The cells received CdCl_2_ stimulation subsequent to *ELAVL1* siRNA transfection. (L) *PEX2* mRNA decay in JEG‐3 cells. Cells were pre‐incubated with ActD and transfected with *ELAVL1* siRNA before being stimulated with CdCl_2_. (M, N) C11‐BODIPY immunostaining of JEG‐3 cells. (O) Levels of PEX2, CAT, and ACOX1 proteins in JEG‐3 cells. *n* = 3–6. ^*^ *P* < 0.05, ^**^ *P* < 0.01 vs. PBS or Ctrl; *^#^P* < 0.05, ^##^ *P* < 0.01 vs. ActD, Cd or ADd.

### 2.7 Prenatal SAH Supplementation Reverses Placental Ferroptosis and FGR Upon Environmental Stress

S‐adenosylhomocysteine (SAH), a methionine cycle intermediate, can reportedly inhibit the activity of METTL14 [51]. To explore the involvement of m6A modification in placental ferroptosis and FGR caused by Cd exposure, we administered SAH intervention to pregnant mice during Cd exposure (Figure 7A). The results indicated that SAH markedly decreased the Cd‐induced rise in FGR occurrence (Figure 7B,C). Relative to the HCd group, the SAH + HCd group displayed elevated measurements for both fetal and placental sizes (Figure 7D–G). TEM analysis demonstrated that SAH reduced ferroptosis‐related mitochondrial phenotypes, such as cristolysis and atrophy, in the placenta under Cd exposure (Figure 7H). According to immunofluorescence analysis, SAH administration markedly decreased Cd‐induced 4‐HNE accumulation in mouse placentae (Figure 7I,J). Furthermore, SAH counteracted both the drop in GSH and the rise in MDA levels in the placenta following Cd exposure (Figure 7K,L). Relative to the HCd group, the SAH + HCd group displayed higher GPX4 and lower 4‐HNE and ACSL4 in the placenta (Figure 7M). We further examined the involvement of m6A modification in lipid deposition and detoxification induced by Cd exposure. Both TEM and immunofluorescence confirmed that SAH administration markedly suppressed lipid droplet accumulation in placentae subjected to Cd exposure (Figure 7N,O and Figure S14A). Moreover, SAH intervention restored the Cd‐induced decline in both H_2_O_2_‐scavenging enzyme levels and β‐oxidation‐related protein levels in the placenta (Figure 7P). We further examined the impact of Cd exposure on PEX2‐mediated pexophagy. Compared to the HCd group, the SAH + HCd group exhibited decreased PEX2 expression (Figure 7Q). TEM results revealed that SAH administration markedly decreased autophagosome numbers in placentae exposed to Cd (Figure S14B). In summary, SAH supplementation reverses environmental stress‐induced FGR via inhibiting pexophagy‐triggered placental ferroptosis.

**FIGURE 7:**
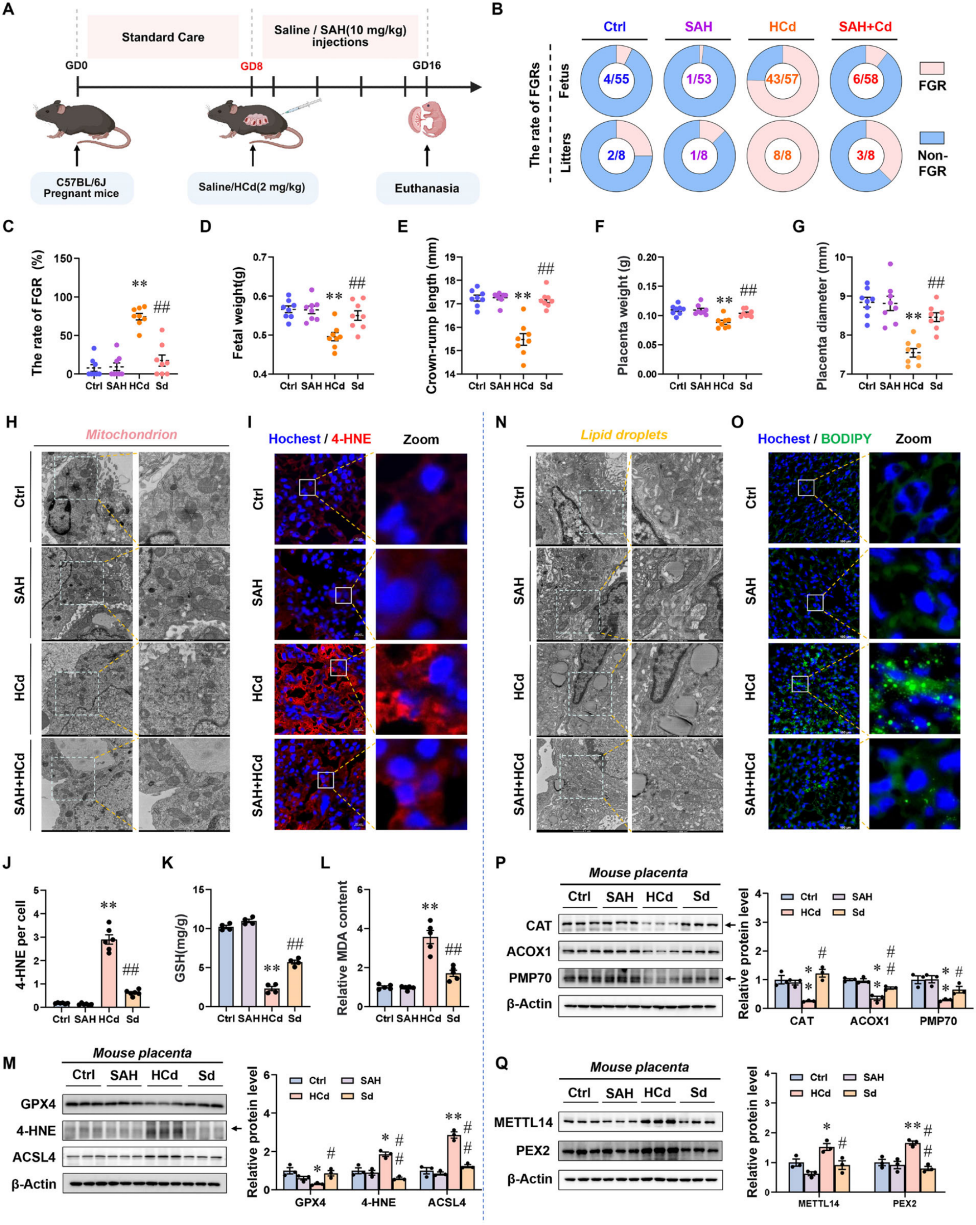
Prenatal SAH supplementation reverses placental ferroptosis and FGR upon environmental stress. (A–Q) On GD 8, pregnant mice from the HCd and SAH+HCd groups received intraperitoneal administration of CdCl_2_ at 2 mg/kg. Pregnant mice from the SAH group and SAH+HCd group underwent intraperitoneal injections of 10 mg/kg SAH on GD 8, 9, 11, 13, and 15. After euthanasia, the placentae were harvested on GD 16. (A) Animal experiment diagram of SAH. (B) Number of litters and number of pups with FGR phenotype. (C) Incidence of fetal growth restriction. (D) Fetal mouse weight. (E) Fetal mouse crown‐rump length. (F) Placental weight. (G) Placental diameter. (H) TEM images of placental cells. (I, J) 4HNE immunostaining of placentae. (K) Mouse placental GSH content. (L) Mouse placental MDA content. (M) Levels of GPX4, 4‐HNE, and ACSL4 in placentae. (N) TEM images of placental cells. (O) BODIPY immunostaining of placentae. (P) Levels of ACOX1, CAT, and PMP70 in placentae. (Q) Levels of PEX2 and METTL14 in placentae. *n* = 3–8. ^*^ *P* < 0.05, ^**^ *P* < 0.01 vs. Ctrl; *^#^P* < 0.05, ^##^ *P* < 0.01 vs. HCd.

### 2.8 Placental Pexophagy is Positively Correlated With Human Placental Ferroptosis Upon Environmental Stress

This study described how prenatal Cd exposure influences m6A modification, pexophagy, placental ferroptosis, and fetal growth. The results confirmed that prenatal Cd exposure activated PEX2‐dependent pexophagy, thereby triggering placental ferroptosis and FGR by promoting m6A modification. Interestingly, the Cd‐induced decrease in GPX4 and increases in PEX2, PEX5, and METTL14 were also observed in placentae exposed to high temperature, another environmental stressor (Figure S15A–E). We subsequently conducted a human case‐control study to validate the association between PEX2‐dependent pexophagy and m6A modification with FGR. According to birth weight, placentas were categorized into AGA or SGA groups (Figure 8A). SGA placentae exhibited elevated pexophagy levels relative to AGA placentae, as evidenced by the increased co‐localization of LC3B and PEX5 (Figure 8B,C). In the SGA placentae, higher PEX2 expression and lower CAT expression were also observed (Figure 8D–F). Furthermore, SGA placentae exhibited higher levels of METTL14 and ELAVL1 (Figure 8G). Immunofluorescence revealed that the SGA group had an increased number of lipid droplets (Figure 8H,I). To further confirm whether Cd exposure induced PEX2‐dependent pexophagy and placental ferroptosis in human placentae, human primary placental trophoblasts were isolated. Cd exposure elevated PEX2 levels while reducing CAT, PMP70, and GPX4 levels in human primary placental trophoblasts (Figure 8J–L). Finally, PEX2‐deficient human primary placental trophoblast cells were generated to explore how pexophagy influences ferroptosis induced by Cd exposure. PEX2 deficiency effectively blocked the decline in CAT and GPX4 levels triggered by Cd exposure in human primary placental trophoblast cells (Figure 8M). In conclusion, PEX2‐dependent pexophagy drives environmental stress‐induced placental ferroptosis and FGR in human.

**FIGURE 8:**
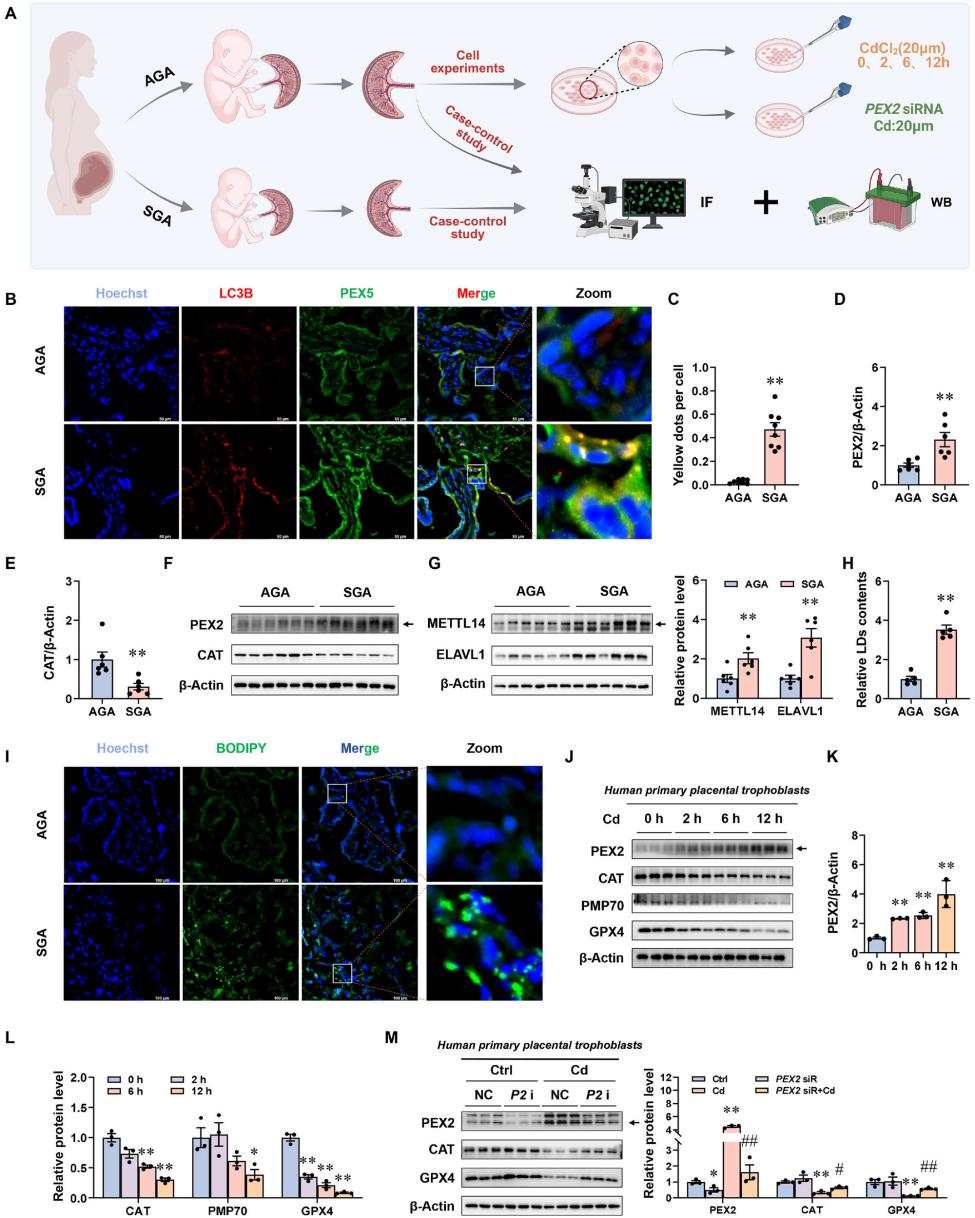
Placental pexophagy is positively correlated with human placental ferroptosis upon environmental stress. (A) Flowchart outlining the collection of human placenta samples in the case‐control study. (B, C) Immunofluorescent detection of LC3B and PEX5 in human placentae. (D–F) Levels of PEX2 and CAT in human placentae. (G) Levels of METTL14 and ELAVL1 in human placentae. (H, I) BODIPY immunofluorescent analysis of human placentae. (J–L) Levels of PEX2, CAT, PMP70, and GPX4 in human primary placental trophoblast cells. (M) Levels of PEX2, CAT, and GPX4 in human primary placental trophoblast cells. The cells received CdCl_2_ stimulation subsequent to *PEX2* siRNA transfection. *n* = 3–6. ^*^ *P* < 0.05, ^**^ *P* < 0.01 vs. AGA, 0 h or Ctrl; *^#^P* < 0.05, ^##^ *P* < 0.01 vs. Cd.

## 3 Discussion

Herein, we sought to elucidate the function of placental ferroptosis in environmental stress and its underlying mechanisms. The study revealed the following innovative findings: (1) Prenatal environmental stressor Cd impairs fetal growth by inducing placental ferroptosis. (2) The activation of PEX5‐dependent pexophagy serves as the primary mediator of this ferroptotic process. (3) Environmental Cd significantly upregulates PEX2 expressison, thereby driving PEX5‐mediated pexophagy in the placenta. (4) These elevated PEX2 levels are fundamentally promoted by Cd‐induced m6A modifications. Building upon the above findings, it can be inferred that prenatal environmental stress exposure promotes m6A modification, thereby activating PEX2‐dependent pexophagy and causing placental ferroptosis and FGR.

The incidence of FGR is as high as 10%, accounting for approximately 30% of the total global perinatal mortality [1]. Although the contribution of environmental exposure to FGR remains unclear, both population‐based and animal studies indicate that environmental stress is an important risk factor for FGR [7, 46, 52, 53]. Prior research further demonstrated that prenatal environmental stress exposure can result in FGR by triggering placental programmed cell death [54, 55, 56]. Ferroptosis, which is driven by the accumulation of lipid peroxides, is an iron‐dependent form of regulated cell death [57]. In this study, placental lipid peroxidation levels were negatively associated with fetal birth weight as well as placental weight. SGA placentae exhibited higher levels of ferroptosis when compared to AGA placentae. These findings suggested that placental ferroptosis may be involved in impaired fetal growth. However, whether ferroptosis contributes to environmental stress‐induced FGR remains unclear. We further established an FGR animal model by exposing pregnant animals to the environmental stressor Cd to explore how ferroptosis contributes to FGR caused by environmental stress. Human studies in China and Egypt reported serum Cd concentrations of 0.044–8.077 and 0.4–2.2 µg/L in pregnant women, respectively [46, 47]. A Nigerian case‐control study reported a serum Cd concentration of approximately 3.37 µg/L among mothers with low‐birth‐weight infants [48]. In this study, maternal serum Cd levels were 1.34 ± 0.09 µg/L in the LCd group and 2.30 ± 0.05 µg/L in the HCd group, overlapping with the ranges reported in the above human studies. In the Cd‐induced mouse FGR model, placental ferroptosis was significantly increased. Notably, supplementation with Fer‐1 reversed environmental stressor Cd‐induced FGR by inhibiting placental ferroptosis. These findings identify that placental ferroptosis may be a key mechanism underlying environmental stress‐induced FGR.

Excessive accumulation of lipid peroxides is the primary mechanism of placental ferroptosis [58, 59]. In this study, targeted oxidized lipidomics showed that the environmental stressor Cd increased placental oxidized lipids mainly derived from VLCFAs and LCFAs. Peroxisomes maintain cellular lipid metabolic homeostasis and redox balance by specifically degrading VLCFAs and scavenging H_2_O_2_^25^. It has been reported that peroxisomal dysfunction leads to the accumulation of oxidized VLCFAs [60]. The environmental stressor Cd decreased levels of peroxisomal proteins ABCD2 and PMP70 without altering the expression of placental PGC1α, a positive regulator promoting lysosome biogenesis. Further supported by the accelerated degradation of ABCD2 in JEG‐3 cells, we speculated that the reduction of peroxisomal proteins was mainly attributable to enhanced degradation rather than reduced biogenesis. Pexophagy refers to the process by which lysosomes target peroxisomes for degradation, leading to the loss of peroxisomal proteins [61]. Further data indicated that Cd‐induced reduction in placental peroxisomal proteins was closely associated with enhanced pexophagy, as evidenced by the increased autophagosome formation, enhanced co‐localization of LC3B‐II with PEX5, and the ability of CQ to inhibit the loss of peroxisomal proteins. Previous studies on the mechanisms of ferroptosis have mainly focused on ferritinophagy and ACSL4‐mediated fatty acid metabolism [62, 63], whereas the role of pexophagy in ferroptosis remains to be elucidated. It has been reported that the buildup of monoubiquitinated PEX5 was a key initiating event in pexophagy [64]. Placenta‐specific *Pex5* knockdown alleviated environmental Cd‐induced placental ferroptosis and FGR, suggesting that PEX5‐dependent pexophagy promotes this process. Furthermore, the activation of placental ferroptosis and pexophagy in SGA placentae and in environmental Cd‐treated human placental primary trophoblast cells suggested this mechanism was also relevant to human FGR. Overall, the above data indicated that prenatal environmental stress induced placental ferroptosis by activating pexophagy.

Peroxisomes play a central role in maintaining lipid metabolism and redox homeostasis. Specifically, peroxisomes scavenge H_2_O_2_ through CAT and degrade VLCFAs via β‐oxidation enzymes ACAA1 and ACOX1, thereby preventing the accumulation of lipid peroxides [65]. In this study, we observed that the environmental stressor Cd simultaneously disrupted both of the above protective functions. To examine the involvement of pexophagy in environmental stress‐induced inhibition of β‐oxidation and H_2_O_2_ clearance, we generated PEX5‐deficient JEG‐3 cells. Importantly, PEX5 deficiency attenuated the Cd‐induced reductions in ACOX1 and CAT expression while alleviating intracellular ROS and lipid droplet accumulation in JEG‐3 cells. Consistently, placenta‐specific *Pex5* knockdown also preserved peroxisomal β‐oxidation and antioxidant capacity, thereby attenuating Cd‐induced H_2_O_2_ and lipid accumulation in mouse placentae. These data indicated that the environmental stressor Cd activated PEX5‐dependent pexophagy to promote placental lipid peroxidation. Mechanistically, the environmental stressor Cd activated pexophagy to reduce ACAA1 and ACOX1, which promoted the accumulation of lipid substrates susceptible to peroxidation. Meanwhile, Cd decreased CAT levels, thereby inhibiting H_2_O_2_ clearance to exacerbate placental oxidative stress. Collectively, pexophagy mediated environmental stress‐induced placental ferroptosis by elevating lipid and ROS levels.

PEX2, PEX10, and PEX12, three E3 ubiquitin ligases, promote monoubiquitination of PEX5 to activate pexophagy [66]. Although the placental mRNA levels of all three factors were elevated following the environmental stressor Cd, *Pex2* mRNA exhibited the greatest increase, accompanied by upregulated PEX2 protein expression in mouse placentae and human trophoblasts. These findings suggested that PEX2 may serve as a major regulator of placental pexophagy in response to environmental Cd. To examine the involvement of PEX2 in environmental stress‐triggered placental pexophagy, we constructed PEX2‐deficient human JEG‐3 cells. PEX2‐deficient cells exhibited elevated levels of PMP70, ACOX1, and CAT, together with reduced ROS and lipid droplet accumulation under environmental Cd. Subsequently, we investigated the effect of PEX2 deficiency on placental ferroptosis upon environmental stress. PEX2 deficiency attenuated Cd‐induced multiple features of ferroptosis, including GPX4 reduction, 4‐HNE and ACSL4 elevation, lipid oxidation, and Fe^2^^+^ accumulation in human JEG3 cells. Consistently, PEX2 deficiency inhibited the degradation of CAT and GPX4 induced by environmental Cd in human placental primary trophoblast cells. Besides, PEX2 levels were increased in SGA placentae and Cd‐treated human placental primary trophoblast cells, further suggesting the relevance of this pathway to human placental pexophagy and ferroptosis. In summary, prenatal environmental stress induced placental ferroptosis through activating PEX2‐dependent pexophagy.

m6A methylation, the most prevalent internal mRNA modification, is involved in regulating post‐transcriptional regulation of gene expression and autophagy [67, 68]. Previous studies have elucidated the roles of m6A modification in ER‐phagy and mitophagy [45, 69], whereas its involvement in environmental stress‐induced pexophagy remains unclear. Environmental stressor Cd exposure significantly enhanced the stability of *PEX2* mRNA in JEG‐3 cells, suggesting that PEX2 increase is post‐transcriptionally regulated. It is well‐established that m6A modification can enhance mRNA stability, thereby promoting protein expression [70]. The increases in METTL14 expression, global m6A levels, and m6A‐methylated *Pex2* mRNA suggested that METTL14‐dependent m6A modification might play a key role in promoting placental PEX2 expression under prenatal Cd exposure. To determine whether METTL14‐dependent m6A modification contributes to the upregulation of PEX2 induced by environmental Cd, we constructed METTL14‐deficient JEG‐3 cells. The findings revealed that METTL14 deficiency accelerated *PEX2* mRNA degradation, thereby reversing PEX2‐dependent pexophagy and ferroptosis under environmental Cd. Reader proteins specifically recognize m6A‐methylated mRNA to regulate the fate of RNA [71]. According to the RM2Target website, ELAVL1 was identified as a potential reader protein of *PEX2* mRNA. ELAVL1 deficiency promoted the degradation of *PEX2* mRNA and attenuated PEX2‐dependent pexophagy, which in turn inhibited placental ferroptosis induced by environmental Cd. Therefore, METTL14‐mediated m6A modification stabilized *PEX2* mRNA in an ELAVL1‐dependent manner, thereby activating placental pexophagy under environmental stress.

SAH, a methionine cycle intermediate, significantly inhibits the enzymatic activity of METTL14 [72]. Previous studies have indicated that SAH could inhibit environmental stress‐induced m6A modification in the placenta [45]. In vitro findings demonstrated that m6A modification promoted pexophagy‐mediated ferroptosis in placental trophoblasts, whereas its therapeutic significance in environmental Cd‐induced FGR remains unclear. To investigate the therapeutic potential of inhibiting m6A modification in environmental Cd‐induced placental ferroptosis and FGR, pregnant mice were treated with SAH. SAH treatment reversed the environmental Cd‐induced reduction in fetal and placental sizes, SAH treatment reversed the environmental Cd‐induced reductions in fetal and placental sizes, indicating a protective effect against FGR. Furthermore, SAH treatment attenuated Cd‐induced molecular and ultrastructural features of placental ferroptosis, including reduced GPX4 and GSH levels, elevated ACSL4, 4‐HNE, and MDA levels, mitochondrial damage, and lipid droplet accumulation. Relative to the HCd group, the SAH + HCd group showed lower PEX2 expression and higher expression of CAT, ACOX1, and PMP70, suggesting that SAH blocks PEX2‐dependent pexophagy under Cd exposure. Collectively, SAH treatment inhibited PEX2‐dependent pexophagy, thereby alleviating placental ferroptosis and FGR under environmental stress. These findings support the in vivo therapeutic potential of targeting m6A modification in environmental stress‐induced FGR.

This study has three main strengths. First, the findings from human placentae, primary trophoblasts, cell lines, and animal models were validated through genetic and pharmacological interventions. Second, Cd exposure served as the primary model, with additional validation in a high‐temperature exposure model, suggesting that the mechanism may extend to other environmental stressors. Third, this study identified an m6A‐PEX2‐pexophagy axis driving placental ferroptosis and confirmed the potential value of SAH intervention in alleviating environmental stress‐induced FGR. The main limitations of this study are as follows: First, although the mechanistic findings were based mainly on a Cd exposure model and further validated in a high‐temperature exposure model, future studies should validate these findings in models of other environmental stressors. Second, this study used SGA as a proxy for FGR, but SGA is a statistical threshold and does not fully equate to pathologically defined FGR. Third, the Cd concentrations used in vitro were higher than those found in human blood, and future studies should employ lower‐dose exposure regimens.

In conclusion, prenatal environmental stress exposure promoted m6A modification, driving PEX2‐dependent pexophagy and ultimately causing placental ferroptosis and FGR. Our study provides new insights into environmental stress‐induced placental cell death, and placental pexophagy may serve as a therapeutic target for FGR and the prevention of fetal‐origin adult diseases.

## 4 Materials and Methods

### 4.1 Chemicals and Reagents

The source and batch number of the main antibodies are provided in Tables S1 and S2.

### 4.2 Animal Experiments

The humane handling standards were established by the Experimental Animal Science Association of Anhui Medical University (Ethical approval number LLSC20242085). Male and female C57BL/6J mice (8 weeks old) and CD‐1 mice (8 weeks old) were provided by Beijing Vital River. All mice were housed under standard conditions with ad libitum access to food and water and were acclimated for two weeks before the study. Cadmium chloride (CdCl_2_) was diluted in saline to prepare working solutions at concentrations of 0.1, 0.2, and 0.45 mg/mL. Ferrostatin‐1 stock (Fer‐1; 10 mg/mL in dimethyl sulfoxide) was diluted with saline to 0.2 mg/mL as the working solution. S‐adenosylhomocysteine stock (SAH; 100 mg/mL in dimethyl sulfoxide) was diluted with saline to 1 mg/mL as the working solution. LV5‐*Ctrl* shRNA and LV5‐*Pex5* shRNA were diluted with saline to prepare working solutions at a concentration of 1 × 10^8^ TU/mL.

To assess the contribution of prenatal environmental stress on FGR, pregnant mice received intraperitoneal administration of varying doses of CdCl_2_ on GD 8. C57BL/6J pregnant mice from the low dose group (LCd) and the high dose group (HCd) were given 1 and 2 mg/kg of CdCl_2_, respectively, while the same volume of normal saline was administered to the control group. All mice were euthanized on GD 16 following anesthesia with Avertin, and the placentae were collected for subsequent analysis. Cd significantly reduced fetal sizes and increased the proportion of fetuses below the 10th percentile of the control weight distribution. Based on these criteria, we defined this model as Cd exposure‐induced FGR.

Next, to evaluate the role of placental ferroptosis in FGR induced by environmental stress, pregnant mice received the ferroptosis‐specific inhibitor Fer‐1 30 min before HCd administration. C57BL/6J pregnant mice in the HCd group and Fer‐1+HCd group were administered CdCl_2_ intraperitoneally at a dose of 2 mg/kg on GD 8. Mice in the Fer‐1 group and Fer‐1+HCd group received intraperitoneal injections of Fer‐1 (2 mg/kg) on GD 8, GD 9, GD 11, GD 13, and GD 15. All mice were euthanized on GD 16 following anesthesia with Avertin, and the placentae were collected for subsequent analysis.

To explore how pexophagy activation contributes to placental ferroptosis induced by maternal environmental stress during gestation, placental *Pex5* was knocked down via placental spot injection of LV5 *Pex5* shRNA. CD‐1 pregnant mice from the HCd group and LV5 *Pex5*+HCd group received intraperitoneal administration of CdCl_2_ with a dose of 2 mg/kg on GD 8. On GD 12, pregnant mice were anesthetized and underwent surgical procedures. According to the group assignment, LV5 *Ctrl* shRNA or LV5 *Pex5* shRNA was injected into the placenta, followed by suturing. After surgery, mice were placed in an incubator and, once fully conscious and able to move spontaneously, were returned to their cages. All pregnant mice were euthanized on GD 16, and the placentae were collected for subsequent analysis.

Meanwhile, to examine the involvement of m6A modification in placental PEX2‐dependent pexophagy induced by maternal environmental stress, C57BL/6J pregnant mice received regular intraperitoneal injections of SAH (10 mg/kg). Pregnant mice from the HCd group and SAH+HCd (Sd) group were administered CdCl_2_ intraperitoneally at a dose of 2 mg/kg on GD 8. In addition, mice in the SAH and Sd groups received intraperitoneal SAH injections on GD 8, 9, 11, 13, and 15. All mice were euthanized on GD 16 following anesthesia with Avertin, and the placentae were collected for subsequent analysis.

To verify whether environmental stress induces placental ferroptosis via pexophagy, we employed heat exposure as another environmental stressor and randomly assigned CD‐1 pregnant mice to control (22°C–24°C) and two heat‐stressed groups under different conditions. From GD 8 to GD 18, mice in the heat stress groups were placed in an incubator at either low‐temperature heat stress (35°C, HT‐L) or high‐temperature heat stress (38°C, HT‐H) for 2 h per day at the same time each day. Both temperatures are above the thermoneutral zone for mice, ensuring that both conditions represent thermal stress. On GD18, all mice were euthanized, and the placentae were collected for further analysis.

### 4.3 Construction of Placenta‐specific *Pex5* Knockdown Mice

Following a previously described method, a placental *Pex5* knockdown mouse model was established via placental spot injection [45]. On GD 8, pregnant mice in the HCd and LV5‐*Ctrl* shRNA + Cd groups received intraperitoneal injections of CdCl_2_ (2 mg/kg). On GD 12, mice were anesthetized and administered meloxicam (2 mg/kg) subcutaneously. The uteruses were then surgically exposed and removed from both sides of the pregnant mice. Using a sterile 34 G Hamilton needle, 3 µL lentiviral vectors (LV5‐*Ctrl* shRNA or LV5‐*Pex5* shRNA, 1 × 10^8^ TU/mL) were injected into each placenta. The uterus was then returned to its original position. To prevent postoperative infection, 1 mL of PBS containing 100 mg/kg cefazolin was injected intraperitoneally. The abdominal incision was sutured, and the mice were placed in a temperature‐controlled incubator until recovery, after which they were returned to their respective cages.

### 4.4 Population Case‐Control Study

Human placental samples were sourced from the Fourth Affiliated Hospital of Anhui Medical University. The case‐control study was approved by the Biomedical Ethics Committee of Anhui Medical University (Ethics Approval Number: KYxM‐202307‐013). Placental samples were collected within 20 min after delivery to ensure tissue integrity. Neonatal and placental weights were recorded simultaneously. Placental tissue samples (2 cm × 2 cm) were collected; portions were fixed in 4% paraformaldehyde for immunofluorescence analysis, while other fresh samples were snap‐frozen and stored at −80°C for molecular analyses and MDA measurement. According to the World Health Organization (WHO), SGA is defined as a birth weight below the 10th percentile for the corresponding gestational age [73]. The International Society of Ultrasound in Obstetrics and Gynecology (ISUOG) defines FGR as the failure to achieve intrinsic growth potential, which is commonly evaluated by integrating fetal size, growth velocity, and Doppler parameters [74]. A previous guideline indicated that SGA may serve as a surrogate phenotype for FGR in the absence of serial ultrasound data [75]. Based on established diagnostic criteria, placentae were classified as AGA and SGA placentae [76]. After controlling for matching factors such as maternal age, BMI, and gestational week, a total of 114 matched placental samples were included in the present study. Demographic and clinical characteristics of these samples are summarized in Table S6.

### 4.5 Cell Lines and Experiments

Human JEG‐3 cells and mouse LZ trophoblast cells are both trophoblast‐lineage cells with similar functions and are extensively used to study the toxicity of environmental contaminants on placental trophoblasts [5, 77, 78]. Human JEG‐3 cells were sourced from the Chinese Academy of Sciences (Shanghai, China). JEG‐3 cells were cultured at 37°C in a humidified 5% CO_2_ incubator. Based on the previous study, JEG‐3 cells were treated with 20 µm CdCl_2_ [5, 79]. The in vitro experiments consisted of eight parts: (1) To determine the dose‐dependent effect of Cd on placental ferroptosis, JEG‐3 cells were treated with Cd at 0, 0.05, 1, and 20 µm for 12 h. (2) To determine the time‐dependent effects of Cd on placental ferroptosis and pexophagy, JEG‐3 cells were exposed to 20 µm CdCl_2_ for 0, 2, 6, and 12 h. (3) To investigate the effect of environmental stress on the degradation rate of peroxisomal proteins, JEG‐3 cells were co‐treated with the translation inhibitor CHX (20 µg/mL) and CdCl_2_ for 0, 3, and 6 h. (4) To determine whether environmental stress induces pexophagy, JEG‐3 cells were pretreated with chloroquine (CQ) for 1 h, followed by exposure to CdCl_2_ for 12 h. (5) The role of pexophagy in environmental stress‐mediated placental ferroptosis was evaluated by transfecting *PEX5* siRNA into JEG‐3 cells. (6) To validate the role of PEX2‐dependent pexophagy in environmental stress‐induced ferroptosis in the placenta, JEG‐3 cells were subjected to *PEX2* siRNA transfection. (7) JEG‐3 cells were exposed to CdCl_2_ and ActD (0.5 µg/mL) for 0, 3, and 6 h to evaluate Cd‐induced changes in *PEX2* mRNA stability and degradation. (8) To investigate the role of m6A modification in activating PEX2‐dependent pexophagy under environmental stress, JEG‐3 cells were transfected with *METTL14* siRNA or *ELAVL1* siRNA, respectively.

### 4.6 Cd Measurement

To determine Cd concentrations in mouse serum, approximately 0.1 mL of serum was transferred into a digestion vessel and diluted 1:1 (v/v) with 1% HNO_3_, followed by thorough mixing. Standard working solutions were prepared by diluting the Cd stock solution with 1% HNO_3_ to final concentrations of 0.0, 0.5, 1.0, 2.0, and 3.0 µg/L. Subsequently, the absorbance of standards and serum samples was measured using graphite furnace atomic absorption spectrophotometry (GFAAS, TAS‐990; Purkinje General Instrument Co., Ltd, Beijing, China). A calibration curve of absorbance vs. concentration was constructed based on the standard working solutions, and the Cd concentration in each serum sample was calculated from this curve.

### 4.7 Isolation of Primary Human Placental Trophoblast Cells

Human placentas were collected within 20 min after delivery and washed with pre‐chilled physiological saline. During tissue processing, blood vessels, heavily calcified regions, and the amniotic membrane were removed. The remaining placental tissue was minced and digested in DMEM medium containing digestive enzymes at 37°C under shaking conditions. The digested tissue was then filtered to retain the main chorionic villus structures. Cell suspensions were filtered through a 300‐mesh sieve and collected by centrifugation. Cell separation was performed using a Percoll gradient, with gradient layers increasing from 5% to 65% (in 5% increments), followed by centrifugation at 1000 g for 10 min. Primary human placental trophoblast cells were harvested from the 45%–40% interface of the gradient. After 24 h of stable culture in complete DMEM medium, the cells were subjected to the indicated treatments.

### 4.8 Isolation of Primary Cells From the Mouse Placenta

Primary mouse placental trophoblast cells were isolated as described previously [80]. Briefly, after euthanizing mice on GD 16, the placentas were removed and washed with pre‐chilled physiological saline to remove blood and impurities. Subsequently, the mouse placenta was minced on ice, and the digestion solution was added. The tissue was then digested on a 37°C constant‐temperature shaker. Post‐digestion, cells were filtered through a 70 µm mesh and washed with DMEM. Cells were then separated using Percoll gradient centrifugation. After fractionation, the collected cells were washed again and seeded into complete culture medium for subsequent experiments.

### 4.9 Hematoxylin and Eosin (H&E) Staining

Mouse placental tissues were fixed in 4% paraformaldehyde at 4°C for 24 h, dehydrated through graded ethanol series and xylene, and embedded in paraffin. The tissues were sectioned into 5‐µm‐thick sections, which were then deparaffinized, rehydrated, and stained with HE. The sections were scanned using a whole‐slide scanner (model S202006121, Leica Microsystems, Germany), and the area ratio of the LZ to the JZ was quantitatively analyzed using ImageJ software.

### 4.10 Immunoblotting

Lysates of placental and cell samples were prepared using RIPA buffer containing 1% PMSF. Protein concentration was quantified using a BCA Protein Assay Kit (Thermo Fisher, Catalog No. 23225). After mixing with loading buffer, the samples were denatured at 100°C for 10 min. Proteins were separated by SDS‐PAGE using gels of appropriate concentration and subsequently electrotransferred onto PVDF membranes. Next, the membranes were blocked in 5% skim milk, then probed with primary antibodies at room temperature for 1.5 h or at 4°C overnight. After washing with TBST, membranes were incubated with secondary antibodies for 1.5 h at room temperature. Protein signals were detected using ECL reagents (K‐12043, Advansat) on a Bio‐Rad imaging system (Model TY2019043988, United States). Band analysis was conducted using IPWIN60 software.

### 4.11 High‐throughput Target Detection of Oxidized Lipids in Mouse Placentae

All pretreatment steps were performed on ice. Each mouse placental tissue sample (25 mg) was weighed and placed into a tube containing two small steel homogenization beads. Then, 600 µL of extraction solution was added, and the sample was vortexed for 30 s. Homogenization was performed using a tissue homogenizer, followed by incubation in an ice‐water bath and two rounds of sonication (5 min per round). The extraction solvent was formulated by mixing n‐hexane and ethyl acetate in a 2:3 volume ratio. Each sample received a 750 µL aliquot of the extraction solvent, followed by a 5 min shake. After centrifugation (3500 rpm, 5 min, 4°C), the top layer (organic phase) was collected. This extraction step (addition of solvent, shaking, centrifugation, and collection) was repeated once, and the two resulting supernatants were pooled together and dried under nitrogen flow. The residue was reconstituted in 100 µL of 30% acetonitrile and centrifuged (12 000 rpm, 15 min, 4 °C). The resulting clear supernatant was used for UHPLC‐MS/MS analysis. In parallel, standard solutions were generated and run under comparable instrument conditions.

### 4.12 Real‐Time RT‐PCR

Total RNA was extracted from mouse placental tissues or JEG‐3 cells using TRIzol reagent on ice, followed by chloroform extraction. Using the Transcriptor First Strand cDNA Synthesis Kit (Roche), RNA was reverse transcribed into cDNA. Quantitative real‐time PCR was performed using the Lightcycler480 system (Roche) to measure target gene mRNA expression levels, with 18S rRNA as the reference gene. The ΔCT method was utilized for comparative analysis of target gene mRNA levels. Primer sequences are listed in Table S3.

### 4.13 Immunofluorescence

After fixation in 4% paraformaldehyde, fresh mouse placental tissues were dehydrated in 30% sucrose solution. The dehydrated tissues were then embedded and sectioned into 5 µm frozen sections using a cryostat. For cell samples, human JEG‐3 cells were seeded onto coverslips in a 12‐well culture plate and cultured until an appropriate confluency was reached. The cells were then subjected to environmental stressors, washed, and fixed with 4% paraformaldehyde solution for 15 min. To minimize non‐specific binding, tissue sections and coverslips were blocked with PBS containing 10% normal donkey serum at 37°C for 1.5 h. Next, primary antibodies (1:200 dilution) were applied and incubated at 37°C for 2 h. After washing, samples were incubated with the appropriate secondary antibodies (conjugated to Cy3 or Alexa Fluor 488) for 1.5 h at 37°C. Fluorescence signals were captured using a confocal microscope (Leica THUNDER DMi8 or Zeiss LSM880 with Airyscan).

### 4.14 Malondialdehyde (MDA) Measurement

Following the protocol provided in the Lipid Peroxidation MDA Assay Kit (Beyotime, Shanghai, China), 40 mg of fresh mouse placental tissue was weighed and homogenized with 400 µL of pre‐cooled lysis buffer to generate a tissue homogenate. The homogenate was then centrifuged at 12 000 g, and the supernatant was collected. After adding the detection reagent according to the specified ratio, the mixture was thoroughly mixed and heated at 100°C for 15 min. Following cooling to room temperature, the mixture was centrifuged at 1000 g for 10 min, and the resulting supernatant was collected. Absorbance was measured using a microplate reader to calculate the MDA content. Finally, MDA levels were normalized to total protein concentration, which was determined separately, and expressed as MDA content per unit tissue protein weight.

### 4.15 Glutathione (GSH) Measurement

Working solutions and standard solutions were prepared according to the manufacturer's instructions in the GSH and GSSG Assay Kit (Beyotime, Shanghai, China). Next, 50 mg of mouse or human placental tissue was homogenized in 500 µL of deproteinizing solution M. The homogenate was incubated at 4°C for 10 min, then centrifuged at 10 000 g (4°C, 10 min), and the supernatant was collected. Subsequently, add the detection solution in proportion. Finally, absorbance was measured using a microplate reader to determine glutathione levels.

### 4.16 Hydrogen Peroxide (H_2_O_2_) Measurement

According to the protocol supplied in the Hydrogen Peroxide Assay Kit (Beyotime, Shanghai, China), 30 mg of mouse placenta was homogenized in 600 µL of pre‐chilled lysis buffer. The homogenate was then centrifuged, and the supernatant was collected. The supernatant was added to the microplate wells along with the detection reagent, and the mixture was mixed thoroughly. After incubation at room temperature for 30 min, H_2_O_2_ content was determined by measuring absorbance using a microplate reader.

### 4.17 Transmission Electron Microscopy (TEM)

Fresh mouse placenta samples were immediately placed into 1% glutaraldehyde fixative solution pre‐cooled to 4°C. The tissues were then minced into 1 mm^3^ fragments and further fixed in 1% glutaraldehyde. Dehydration was performed with a graded ethanol series (50%–100%), after which the samples were embedded in EPON 812 resin. Ultrathin sections, ranging from 70 to 100 nm in thickness, were cut with a Leica EM UC7 ultramicrotome (Germany). Images were acquired on a Thermo Fisher Scientific Talos L120CG2 TEM after staining with lead citrate.

### 4.18 BODIPY Staining for Lipid Droplet Detection

Live cells or frozen tissue sections were first rinsed twice with PBS solution. Meanwhile, BODIPY 493/503 (MED25592) was diluted to a working concentration immediately prior to use, applied to cells or tissue sections, and incubated at 37°C for 20 min. After incubation, samples were washed with PBS and subsequently stained with Hoechst 33258 for 6 min. Finally, fluorescence images were acquired using a laser confocal microscope (Leica THUNDER DMi8, Germany).

### 4.19 FerroOrange Detection

After JEG‐3 cells were exposed to environmental stress for 12 h, the cells were rinsed twice with PBS. Next, Hoechst 33258 staining solution was applied for 6 min. Subsequently, the cells were washed twice with PBS, then incubated with 1 µmol/L FerroOrange staining solution for 30 min. After incubation, the cells were directly imaged using a confocal laser scanning microscope (Leica THUNDER DMi8, Germany).

### 4.20 ROS Measurement

ROS levels were measured using the DCFH‐DA probe, as previously described. Briefly, JEG‐3 cells were subjected to environmental stress for 12 h and washed twice with PBS. After that, the cells were exposed to 10 µm DCFH‐DA probe for 30 min at 37°C. The samples were then visualized using a confocal microscope (Leica THUNDER DMi8, Germany).

### 4.21 C11‐BODIPY Staining for Oxidized Lipids Detection

C11‐BODIPY (Thermo Fisher Scientific) dye stock solution was diluted at a 1:1000 ratio. The freshly prepared working solution was added to the cells, followed by incubation for 30 min. After incubation, the cells were rinsed three times with PBS. Hoechst 33258 was then applied and incubated for 6 min, followed by three PBS washes. Fluorescence signals were observed using a laser confocal microscope (Leica THUNDER DMi8, Germany). The excitation wavelengths used were 510 nm for oxidized lipids and 590 nm for reduced lipids.

### 4.22 RNA Interference

Cells were cultured in 100‐mm culture dishes until confluence reached approximately 70%. Corresponding siRNAs were transfected using Lipofectamine 3000 transfection reagent (Invitrogen) to achieve target gene knockdown. Following the manufacturer's instructions, serum‐free Opti‐MEM was used to dilute siRNA (as the interference reagent) or Lipofectamine 3000 (as the transfection reagent). After a 5 min standing period, the transfection reagent was gently mixed with the interfering agent and allowed to stand for another 15 min. The culture medium was then replaced with serum‐free Opti‐MEM, and the transfection mixture was added to the cells for 6 h. Subsequently, cells were cultured in complete medium for 42 h to allow gene knockdown. After treatment with CdCl_2_, total RNA and proteins were extracted for subsequent analyses. siRNA sequences are listed in Table S4.

### 4.23 RNA Stability Assay

To evaluate how environmental stress exposure affects *PEX2* mRNA degradation, JEG‐3 cells were co‐treated with 20 µm CdCl_2_ and 0.5 µg/mL ActD (a transcription inhibitor). JEG‐3 cells lacking METTL14/ELAVL1 were treated with 20 µm CdCl_2_ and 0.5 µg/mL ActD to determine the role of these proteins in environmental stress‐evoked *PEX2* mRNA stabilization. Following exposure to Cd for 0, 3, or 6 h, total RNA was extracted, and *PEX2* mRNA levels were quantified using real‐time PCR.

### 4.24 Assessment of m6A Levels

Total RNA was extracted from mouse placental tissues using TRIzol reagent. The m6A RNA methylation status was assessed using the EpiQuik m6A RNA Methylation Quantification Kit (Epigentek). Briefly, each well was preloaded with 80 µL of binding solution and 2 µL of diluted positive control, followed by the addition of 200 ng of RNA or positive control to the respective wells. Capture antibody solution was added sequentially to each well, incubated at room temperature for 60 min, and then rinsed three times with washing buffer. The samples were then treated as follows: incubation with antibody solution (30 min, room temperature), followed by four washes; addition of antibody enhancer, incubation (30 min, room temperature), followed by five washes; and incubation in development solution for 10 min. Absorbance was measured at 450 nm using a SYNERGY4 Multimode Microplate Reader (Biotek, USA). Total m6A levels were calculated based on a standard curve.

### 4.25 MeRIP‐PCR

MeRIP‐qPCR experiments were performed according to previously published protocols [81]. Briefly, mouse placental tissues were lysed with TRIzol reagent, and total RNA was extracted using chloroform‐based phase separation. Next, mRNA was purified from total RNA using the Arraystar Seq‐Star Poly(A) mRNA Purification Kit (Maryland, USA). As a control, 10% of the purified mRNA was retained. For quantification of total m6A in *PEX2* mRNA, the reaction system was supplemented with 2 µg of anti‐m6A polyclonal antibody (Synaptic System, 202003) and then incubated at 4°C for 2 h. Then, the reaction mixture was supplemented with Dynabeads M‐280 Sheep Anti‐Rabbit IgG (Thermo Fisher, 11204D) and incubated for an additional 2 h at 4°C. The beads were subsequently washed sequentially with binding buffer and wash buffer. Elution buffer was added, and the samples were incubated at 50°C for 30 min to release the immunoprecipitated RNA. The resulting m6A‐enriched mRNA was extracted using chloroform. The levels of m6A‐enriched *PEX2* mRNA were quantified using the LightCycler 480 real‐time fluorescent quantitative PCR system. Primer sequences for each locus are provided in Table S5.

### 4.26 Statistical Analysis

Data are presented as mean ± standard error of the mean (SEM). Statistical analyses were performed using SPSS 23.0 software (IBM, USA). Differences between the two groups were assessed using a two‐tailed Student's *t*‐test. For comparisons among multiple groups, one‐way analysis of variance (ANOVA) was applied, followed by Bonferroni post‐hoc tests. A *P*‐value < 0.05 was considered statistically significant.

## Author Contributions

**Xin‐Xin Zhang**: project administration and writing – original draft. **Ye‐Xin Luo**: data curation. **Cheng‐Fang Sun**: formal analysis. **Rui Pan**: validation. **Yu‐Xi Wu**: resources. **Zhi Yuan**: visualization. **Xin‐Run Wang**: visualization. **Xu‐Dong Zhang**: methodology. **Lan‐Lan Wu**: formal analysis. **Xiang‐Yu Yao**: validation. **Zhan‐Dong Song**: validation. **Wei Chang**: methodology. **Tian Wei**: resources. **Pan‐Yuan Cai**: resources. **Tian‐Ci Zhang**: resources. **Yong‐Wei Xiong**: methodology. **De‐Xiang Xu**: validation. **Hua Wang**: writing – review, and editing. **Hua‐Long Zhu**: conceptualization, methodology, resources, writing – review, and editing.

## Funding

This work was supported by the National Natural Science Foundation of China (82304181 and 82273664), the Scientific Research Foundation of Education Department of Anhui Province (2024AH030024, 2022AH050644 and 2025AHGXZK40743), the Research Funds of Center for Big Data and Population Health of IHM (JKS2022016), the Natural Science Foundation of Anhui Province (2008085J38), and the Academic Funding Project for Top Talents in Colleges and Universities (gxbjZD2020059).

## Conflicts of Interest

The authors declare no conflicts of interest.

## Supporting information




**Supporting File**: advs77068‐sup‐0001‐SuppMat.docx.

## Data Availability

The data that supports the findings of this study are available in the supplementary material of this article.

## References

[1] J. G. Martins , J. R. Biggio , and A. Abuhamad , “Society for Maternal‐Fetal Medicine Consult Series #52: Diagnosis and management of fetal growth restriction: (Replaces Clinical Guideline Number 3, April 2012),” American Journal of Obstetrics and Gynecology 223 (2020): B2–B17. 10.1016/j.ajog.2020.05.010 32407785

[2] J. M. Alsweiler , D. L. Harris , J. E. Harding , and C. J. D. McKinlay , “Strategies to Improve Neurodevelopmental Outcomes in Babies at Risk of Neonatal Hypoglycaemia,” The Lancet Child & Adolescent Health 5 (2021): 513–523, 10.1016/S2352-4642(20)30387-4. PMC8528170 33836151

[3] A. K. Oestreich and K. H. Moley , “Developmental and Transmittable Origins of Obesity‐Associated Health Disorders,” Trends in Genetics 33 (2017): 399–407, 10.1016/j.tig.2017.03.008. PMC5875684 28438343

[4] F. Crispi , J. Miranda , and E. Gratacós , “Long‐Term Cardiovascular Consequences of Fetal Growth Restriction: Biology, Clinical Implications, and Opportunities for Prevention of Adult Disease,” American Journal of Obstetrics and Gynecology 218 (2018): S869–S879, 10.1016/j.ajog.2017.12.012. 29422215

[5] H.‐L. Zhu , L.‐M. Dai , Y.‐W. Xiong , et al., “Gestational Exposure to Environmental Cadmium Induces Placental Apoptosis and Fetal Growth Restriction via Parkin‐Modulated MCL‐1 Degradation,” Journal of Hazardous Materials 424 (2022): 127268, 10.1016/j.jhazmat.2021.127268. 34583167

[6] L. Zheng , R. Tang , F. Ahmad , et al., “Circ_0081343 Promotes Autophagy and Alleviates Pyroptosis via PI3 K/AKT/HIF‐1α axis In Hypoxia‐Induced Fetal Growth Restriction of Mice,” Animal cells and systems 29 (2025): 312–324, 10.1080/19768354.2025.2498932. PMC12064109 40353255

[7] H.‐L. Zhu , X.‐T. Shi , X.‐F. Xu , et al., “Environmental Cadmium Exposure Induces Fetal Growth Restriction via Triggering PERK‐Regulated Mitophagy In Placental Trophoblasts,” Environment International 147 (2021): 106319, 10.1016/j.envint.2020.106319. 33348103

[8] X. Chen , J. Li , R. Kang , D. J. Klionsky , and D. Tang , “Ferroptosis: Machinery and Regulation,” Autophagy 17 (2021): 2054–2081, 10.1080/15548627.2020.1810918. PMC8496712 32804006

[9] Y. You , Z. Qian , Y. Jiang , et al., “Insights Into the Pathogenesis of Gestational and Hepatic Diseases: The Impact of Ferroptosis,” Frontiers in Cell and Developmental Biology 12 (2024): 1482838, 10.3389/fcell.2024.1482838. PMC11588751 39600338

[10] C. Park , S. Alahari , J. Ausman , et al., “Placental Hypoxia‐Induced Ferroptosis Drives Vascular Damage in Preeclampsia,” Circulation Research 136 (2025): 361–378, 10.1161/CIRCRESAHA.124.325119. 39846172

[11] S. Yi , H. Wen Xin , Q. Fan , et al., “Lithium Exposure Causes Ferroptosis to Induce Unexplained Miscarriage Through Non‐Canonical MBOAT1 Pathway,” Nature Communications 17 (2025): 365, 10.1038/s41467-025-67026-7. PMC12796209 41381569

[12] Q. Zhang , X. Yuan , X. Luan , et al., “GLUT1 Exacerbates Trophoblast Ferroptosis by Modulating AMPK/ACC Mediated Lipid Metabolism and Promotes Gestational Diabetes Mellitus Associated Fetal Growth Restriction,” Molecular Medicine 30 (2024): 257, 10.1186/s10020-024-01028-x. PMC11660491 39707215

[13] X.‐D. Zhang , J. Sun , X.‐M. Zheng , et al., “Plin4 Exacerbates Cadmium‐Decreased Testosterone Level via Inducing Ferroptosis in Testicular Leydig Cells,” Redox Biology 76 (2024): 103312, 10.1016/j.redox.2024.103312. PMC11387904 39173539

[14] Y.‐T. Lv , T.‐B. Liu , Y. Li , Z.‐Y. Wang , C.‐Y. Lian , and L. Wang , “HO‐1 Activation Contributes to Cadmium‐Induced Ferroptosis in Renal Tubular Epithelial Cells via Increasing the Labile Iron Pool and Promoting Mitochondrial ROS Generation,” Chemico‐Biological Interactions 399 (2024): 111152, 10.1016/j.cbi.2024.111152. 39025289

[15] Y.‐F. Zhang , S. Zhang , Q. Ling , et al., “Activation of Lipophagy Ameliorates Cadmium‐Induced Neural Tube Defects via Reducing Low Density Lipoprotein Cholesterol Levels in Mouse Placentas,” Cell Biology and Toxicology 40 (2024): 35, 10.1007/s10565-024-09885-2. PMC11108957 38771546

[16] H.‐L. Zhu , X.‐T. Shi , X.‐F. Xu , et al., “Melatonin Protects Against Environmental Stress‐Induced Fetal Growth Restriction via Suppressing ROS‐Mediated GCN2/ATF4/BNIP3‐Dependent Mitophagy in Placental Trophoblasts,” Redox Biology 40 (2021): 101854, 10.1016/j.redox.2021.101854. PMC7811044 33454563

[17] D. H. Cho , Y. S. Kim , D. S. Jo , S. K. Choe , and E. K. Jo , “Pexophagy: Molecular Mechanisms and Implications for Health and Diseases,” Molecules and Cells 41 (2018): 55–64. 10.14348/molcells.2018.2245 PMC5792714 29370694

[18] J. Zhang , D. N. Tripathi , J. Jing , et al., “ATM Functions at the Peroxisome to Induce Pexophagy in Response to ROS,” Nature cell biology 17 (2015): 1259–1269, 10.1038/ncb3230. PMC4589490 26344566

[19] J. N. S. Vargas , M. Hamasaki , T. Kawabata , R. J. Youle , and T. Yoshimori , “The Mechanisms and Roles of Selective Autophagy in Mammals,” Nature Reviews Molecular Cell Biology 24 (2023): 167–185, 10.1038/s41580-022-00542-2. 36302887

[20] D.‐H. Cho , Y. S. Kim , D. S. Jo , S.‐K. Choe , and J. E.‐K. Pexophagy , “Molecular Mechanisms and Implications for Health and Diseases,” Molecules and Cells 41 (2018): 55–64. 10.14348/molcells.2018.2245 PMC5792714 29370694

[21] X. Wei , L. Manandhar , H. Kim , et al., “Pexophagy and Oxidative Stress: Focus on Peroxisomal Proteins and Reactive Oxygen Species (ROS) Signaling Pathways,” Antioxidants 14 (2025): 126. 10.3390/antiox14020126 PMC11851658 40002313

[22] R. Vasko , B. B. Ratliff , S. Bohr , et al., “Endothelial Peroxisomal Dysfunction and Impaired Pexophagy Promotes Oxidative Damage in Lipopolysaccharide‐Induced Acute Kidney Injury,” Antioxidants & redox signaling 19 (2013): 211–230, 10.1089/ars.2012.4768. PMC3691927 23088293

[23] S. Kan , Q. Hou , J. Shi , et al., “EHHADH Deficiency Regulates Pexophagy and Accelerates Tubulointerstitial Injury in Diabetic Kidney Disease,” Cell death discovery 10 (2024): 289, 10.1038/s41420-024-02066-4. PMC11180138 38879653

[24] H. R. Waterham , J. Koster , M. S. Ebberink , et al., “Autosomal Dominant Zellweger Spectrum Disorder Caused by de novo Variants in PEX14 Gene,” Genetics in Medicine 25 (2023): 100944, 10.1016/j.gim.2023.100944. 37493040

[25] F. Di Cara , S. Savary , W. J. Kovacs , P. Kim , and R. A. Rachubinski , “The Peroxisome: An up‐and‐Coming Organelle in Immunometabolism,” Trends in Cell Biology 33 (2023): 70–86. 10.1016/j.tcb.2022.06.001 35788297

[26] A. He , J. M. Dean , and I. J. Lodhi , “Peroxisomes as Cellular Adaptors to Metabolic and Environmental Stress,” Trends in Cell Biology 31 (2021): 656–670, 10.1016/j.tcb.2021.02.005. PMC8566112 33674166

[27] R. Vasko and M. S. Goligorsky , “Dysfunctional Lysosomal Autophagy Leads to Peroxisomal Oxidative Burnout and Damage During Endotoxin‐Induced Stress,” Autophagy 9 (2013): 442–444, 10.4161/auto.23344. PMC3590273 23328407

[28] L. Manandhar , R. K. Dutta , P. Devkota , et al., “TFEB Activation Triggers Pexophagy for Functional Adaptation During Oxidative Stress Under Calcium Deficient‐Conditions,” Cell Communication and Signaling 22 (2024): 142, 10.1186/s12964-024-01524-x. PMC10880274 38383392

[29] A. Jin , J. N. Lee , M. S. Kim , et al., “2,2′‐dipyridyl Induces Pexophagy,” Biochemical and Biophysical Research Communications 469 (2016): 941–947, 10.1016/j.bbrc.2015.12.098. 26721431

[30] H. Jiang , Y. Qu , X. Kan , et al., “Pexophagy‐Driven Redox Imbalance Promotes Virus‐Induced Ferroptosis,” Cell Reports 44 (2025): 115783, 10.1016/j.celrep.2025.115783. 40478734

[31] H. Luo , M. G. Kharas , and S. R. Jaffrey , “N6‐Methyladenosine: An RNA Modification as a Central Regulator of Cancer,” Nature Reviews Cancer 26 (2026): 118–136, 10.1038/s41568-025-00889-6. PMC13142248 41360987

[32] Y. An and H. Duan , “The Role of m6A RNA Methylation in Cancer Metabolism,” Molecular Cancer 21 (2022): 14, 10.1186/s12943-022-01500-4. PMC8753874 35022030

[33] H. Yu , J. Zhuang , Z. Zhou , et al., “METTL16 Suppressed the Proliferation and Cisplatin‐Chemoresistance of Bladder Cancer by Degrading PMEPA1 mRNA in a m6A Manner Through Autophagy Pathway,” International Journal of Biological Sciences 20 (2024): 1471–1491, 10.7150/ijbs.86719. PMC10878153 38385084

[34] F. Wang , Q. Liao , Z. Qin , et al., “Autophagy: A Critical Mechanism of N6‐Methyladenosine Modification Involved in Tumor Progression and Therapy Resistance,” Cell Death & Disease 15 (2024): 783, 10.1038/s41419-024-07148-w. PMC11519594 39468015

[35] L. Liu , H. Li , D. Hu , et al., “Insights Into N6‐Methyladenosine and Programmed Cell Death in Cancer,” Molecular Cancer 21 (2022): 32, 10.1186/s12943-022-01508-w. PMC8796496 35090469

[36] H. Fu , C. Chen , Y. Huang , et al., “METTL3 Safeguards Cell Identity and Epigenome of Human Trophoblast Stem Cells,” Nature Communications 17 (2025): 795, 10.1038/s41467-025-67484-z. PMC12823569 41402305

[37] W. Li , Q. Zhang , M. Ni , et al., “Upregulated YTHDC1 Mediates Trophoblastic Dysfunction Inducing Preterm Birth in ART Conceptions Through Enhanced RPL37 Translation,” Cellular and Molecular Life Sciences 82 (2024): 17, 10.1007/s00018-024-05467-x. PMC11671673 39725796

[38] R. Wang , X. Xu , J. Yang , et al., “BPDE Exposure Promotes Trophoblast Cell Pyroptosis and Induces Miscarriage by Up‐Regulating lnc‐HZ14/ZBP1/NLRP3 Axis,” Journal of Hazardous Materials 455 (2023): 131543, 10.1016/j.jhazmat.2023.131543. 37167865

[39] M. Dai , W. Huang , X. Huang , et al., “BPDE, the Migration and Invasion of Human Trophoblast Cells, and Occurrence of Miscarriage in Humans: Roles of a Novel lncRNA‐HZ09,” Environmental Health Perspectives 131 (2023): 17009, 10.1289/EHP10477. PMC9888265 36719213

[40] Y.‐P. Song , J.‐W. Lv , Z.‐C. Zhang , et al., “Effects of Gestational Arsenic Exposures on Placental and Fetal Development in Mice: The Role of Cyr61 m6A,” Environmental Health Perspectives 131 (2023): 97004, 10.1289/EHP12207. PMC10489955 37682722

[41] Z. Chen , Z. Chen , J. Mo , Y. Chen , L. Chen , and C. Deng , “m6A RNA Methylation Modulates Autophagy by Targeting Map1lc3b in Bisphenol A Induced Leydig Cell Dysfunction,” Journal of Hazardous Materials 485 (2025): 136748, 10.1016/j.jhazmat.2024.136748. 39662354

[42] M. Shen , Y. Li , Y. Wang , et al., “N6‐Methyladenosine Modification Regulates Ferroptosis Through Autophagy Signaling Pathway in Hepatic Stellate Cells,” Redox Biology 47 (2021): 102151, 10.1016/j.redox.2021.102151. PMC8495178 34607160

[43] T. Wu , Y. Shao , X. Li , et al., “NR3C1/Glucocorticoid Receptor Activation Promotes Pancreatic β‐Cell Autophagy Overload in Response to Glucolipotoxicity,” Autophagy 19 (2023): 2538–2557, 10.1080/15548627.2023.2200625. PMC10392762 37039556

[44] D. S. Jo , N. Y. Park , and D. H. Cho , “Peroxisome Quality Control and Dysregulated Lipid Metabolism in Neurodegenerative Diseases,” Experimental & Molecular Medicine 52 (2020): 1486–1495, 10.1038/s12276-020-00503-9. PMC8080768 32917959

[45] Y.‐X. Luo , H.‐L. Zhu , B.‐B. Huang , et al., “Placental RTN3L‐dependent ER‐Phagy Contributes to Fetal Testicular Dysplasia Upon Environmental Stress,” Advanced Science 12 (2025): 2500924, 10.1002/advs.202500924. PMC12224940 40285582

[46] H. Wang , L. Liu , Y.‐F. Hu , et al., “Maternal Serum Cadmium Level During Pregnancy and its Association With Small for Gestational Age Infants: A Population‐Based Birth Cohort Study,” Scientific Reports 6 (2016): 22631, 10.1038/srep22631. PMC4776171 26934860

[47] G. Mokhtar , E. Hossny , M. el‐Awady , and M. Zekry , “In Utero Exposure to Cadmium Pollution in Cairo and Giza Governorates of Egypt,” Eastern Mediterranean Health Journal 8 (2002): 254–260. 15339112

[48] E. P. Ikeh‐Tawari , J. I. Anetor , and C.‐D. MA , “Cadmium Level in Pregnancy, Influence on Neonatal Birth Weight and Possible Amelioration by Some Essential Trace Elements,” Toxicology International 20 (2013): 108–112, 10.4103/0971-6580.111558. PMC3702118 23833446

[49] C. Scarpellini , G. Klejborowska , C. Lanthier , B. Hassannia , T. Vanden Berghe , and K. Augustyns , “Beyond Ferrostatin‐1: A Comprehensive Review of Ferroptosis Inhibitors,” Trends in Pharmacological Sciences 44 (2023): 902–916, 10.1016/j.tips.2023.08.012. 37770317

[50] E. Mishima , T. Nakamura , S. Doll , et al., “Recommendations for Robust and Reproducible Research on Ferroptosis,” Nature Reviews Molecular Cell Biology 26 (2025): 615–630, 10.1038/s41580-025-00843-2. 40204928

[51] P. Wang , A. Doxtader Katelyn , and Y. Nam , “Structural Basis for Cooperative Function of Mettl3 and Mettl14 Methyltransferases,” Molecular Cell 63 (2016): 306–317, 10.1016/j.molcel.2016.05.041. PMC4958592 27373337

[52] M. F. Chersich , M. D. Pham , A. Areal , et al., “Associations Between High Temperatures in Pregnancy and Risk of Preterm Birth, Low Birth Weight, and Stillbirths: Systematic Review and Meta‐Analysis,” BMJ 371 (2020): m3811, 10.1136/bmj.m3811. PMC7610201 33148618

[53] J.‐J. Wu , X. Zheng , C. Wu , et al., “Melatonin Alleviates High Temperature Exposure Induced Fetal Growth Restriction via the Gut‐Placenta‐Fetus Axis in Pregnant Mice,” Journal of Advanced Research 68 (2025): 131–146, 10.1016/j.jare.2024.02.014. PMC11785557 38382594

[54] H. Zhang , X. Zha , B. Zhang , et al., “Gut Microbiota Contributes to Bisphenol A‐Induced Maternal Intestinal and Placental Apoptosis, Oxidative Stress, and Fetal Growth Restriction in Pregnant Ewe Model by Regulating Gut‐Placental Axis,” Microbiome 12 (2024): 28, 10.1186/s40168-024-01749-5. PMC10874076 38365714

[55] J. R. Liu , F. N. Wu , S. Lin , et al., “Gestational Zearalenone Causes Fetal Intrauterine Growth Restriction Partially Through Deriving ROS‐Drp1 Mediated Placental PANoptosis,” Ecotoxicology and Environmental Safety 302 (2025): 118636, 10.1016/j.ecoenv.2025.118636. 40712548

[56] C. Zhang , L. Xiao , Z. Fang , et al., “Gestational Exposure to Black Phosphorus Nanoparticles Induces Placental Trophoblast Dysfunction by Triggering Reactive Oxygen Species‐Regulated Mitophagy,” ACS Nano 19 (2025): 16517–16533, 10.1021/acsnano.4c18731. PMC12060646 40264356

[57] S. J. Dixon and J. A. Olzmann , “The Cell Biology of Ferroptosis,” Nature Reviews Molecular Cell Biology 25 (2024): 424–442, 10.1038/s41580-024-00703-5. PMC12187608 38366038

[58] B. Wiernicki , H. Dubois , Y. Y. Tyurina , et al., “Excessive Phospholipid Peroxidation Distinguishes Ferroptosis From Other Cell Death Modes Including Pyroptosis,” Cell Death & Disease 11 (2020): 922, 10.1038/s41419-020-03118-0. PMC7591475 33110056

[59] X. Chen , R. Kang , G. Kroemer , and D. Tang , “Organelle‐Specific Regulation of Ferroptosis,” Cell Death & Differentiation 28 (2021): 2843–2856, 10.1038/s41418-021-00859-z. PMC8481335 34465893

[60] F. Di Cara , P. Andreoletti , D. Trompier , et al., “Peroxisomes in Immune Response and Inflammation,” International Journal of Molecular Sciences 20 (2019): 3877. 10.3390/ijms20163877 PMC6721249 31398943

[61] K. Germain and P. K. Kim , “Pexophagy: A Model for Selective Autophagy,” International Journal of Molecular Sciences 21 (2020): 578, 10.3390/ijms21020578.

[62] J. Liang , Y. Liao , P. Wang , et al., “Ferroptosis Landscape in Prostate Cancer From Molecular and Metabolic Perspective,” Cell Death Discovery 9 (2023): 128, 10.1038/s41420-023-01430-0. PMC10105735 37061523

[63] T. Xu , Z. Xie , Y. Zhao , et al., “Melatonin Impedes NCOA4/ACSL4‐Dependent Ferroptosis by Targeting STING to Ameliorate Diabetic Cognitive Dysfunction,” Bioorganic Chemistry 169 (2026): 109357, 10.1016/j.bioorg.2025.109357. 41418601

[64] M. Nordgren , T. Francisco , C. Lismont , et al., “Export‐Deficient Monoubiquitinated PEX5 Triggers Peroxisome Removal in SV40 Large T Antigen‐Transformed Mouse Embryonic Fibroblasts,” Autophagy 11 (2015): 1326–1340, 10.1080/15548627.2015.1061846. PMC4590649 26086376

[65] R. J. A. Wanders , M. Baes , D. Ribeiro , S. Ferdinandusse , and H. R. Waterham , “The Physiological Functions of Human Peroxisomes,” Physiological Reviews 103 (2022): 957–1024, 10.1152/physrev.00051.2021. 35951481

[66] J. C. Farré , S. S. Mahalingam , M. Proietto , and S. Subramani , “Peroxisome Biogenesis, Membrane Contact Sites, and Quality Control,” EMBO Reports 20 (2019): EMBR201846864. 10.15252/embr.201846864 PMC6322382 30530632

[67] L. Ling , T. Hu , C. Zhou , et al., “M6A‐Methylated circRAPGEF5 Drives Lung Adenocarcinoma Progression and Metastasis via IGF2BP2/NUP160‐Mediated Autophagy Suppression,” Molecular Cancer 24 (2025): 192, 10.1186/s12943-025-02399-3. PMC12236038 40629330

[68] Q. Li , Y. Ni , L. Zhang , et al., “HIF‐1α‐Induced Expression of m6A Reader YTHDF1 Drives Hypoxia‐Induced Autophagy and Malignancy of Hepatocellular Carcinoma by Promoting ATG2A and ATG14 Translation,” Signal Transduction and Targeted Therapy 6 (2021): 76, 10.1038/s41392-020-00453-8. PMC7900110 33619246

[69] Y. Liu , G. Li , A. Xiong , et al., “Fine Particulate Matter Exacerbates Asthma by Activating STC2‐Mediated Mitophagy Through METTL3/YTHDF2‐Dependent m6A Methylation,” Journal of Hazardous Materials 495 (2025): 138854, 10.1016/j.jhazmat.2025.138854. 40499413

[70] M. Rossi , N. Banskota , C. H. Shin , et al., “Increased PTCHD4 Expression via m6A Modification of PTCHD4 mRNA Promotes Senescent Cell Survival,” Nucleic Acids Research 52 (2024): 7261–7278, 10.1093/nar/gkae322. PMC11229380 38721764

[71] H. Shi , J. Wei , and C. He , “Where, When, and How: Context‐Dependent Functions of RNA Methylation Writers, Readers, and Erasers,” Molecular Cell 74 (2019): 640–650, 10.1016/j.molcel.2019.04.025. PMC6527355 31100245

[72] P. Wang , K. A. Doxtader , and Y. Nam , “Structural Basis for Cooperative Function of Mettl3 and Mettl14 Methyltransferases,” Molecular Cell 63 (2016): 306–317, 10.1016/j.molcel.2016.05.041. PMC4958592 27373337

[73] “Physical Status: The Use and Interpretation of Anthropometry,” Report of a WHO Expert Committee. World Health Organization Technical Report Series (World Health Organization, 1995), 1–452. 8594834

[74] C. C. Lees , T. Stampalija , A. A. Baschat , et al., “ISUOG Practice Guidelines: Diagnosis and Management of Small‐for‐Gestational‐Age Fetus and Fetal Growth Restriction,” Ultrasound in Obstetrics & Gynecology: The Official Journal of the International Society of Ultrasound in Obstetrics and Gynecology 56 (2020): 298–312, 10.1002/uog.22134. 32738107

[75] E. P. Schlaudecker , F. M. Munoz , A. Bardají , et al., “Small for Gestational Age: Case Definition & Guidelines for Data Collection, Analysis, and Presentation of Maternal Immunisation Safety Data,” Vaccine 35 (2017): 6518–6528, 10.1016/j.vaccine.2017.01.040. PMC5710996 29150057

[76] C. C. Lees , R. Romero , T. Stampalija , et al., “The Diagnosis and Management of Suspected Fetal Growth Restriction: An Evidence‐Based Approach,” American Journal of Obstetrics and Gynecology 226 (2022): 366–378, 10.1016/j.ajog.2021.11.1357. PMC9125563 35026129

[77] A. Ghosh , R. Kumar , R. P. Kumar , et al., “The GATA Transcriptional Program Dictates Cell Fate Equilibrium to Establish The Maternal–Fetal Exchange Interface and Fetal Development,” Proceedings of the National Academy of Sciences 121 (2024): 2310502121, 10.1073/pnas.2310502121. PMC10895349 38346193

[78] C. A. McConkey , E. Delorme‐Axford , C. A. Nickerson , et al., “A Three‐Dimensional Culture System Recapitulates Placental Syncytiotrophoblast Development and Microbial Resistance,” Science Advances 2 (2016): 1501462, 10.1126/sciadv.1501462. PMC4783126 26973875

[79] R. Ramakrishnan and A. C. Daly , “Revisiting ISO 10993‐5 In Vitro Cytotoxicity Standard Tests for Evaluation of Extracellular Matrix‐Based Biomaterials,” International Journal of Biomaterials 2026 (2026): 7395612, 10.1155/ijbm/7395612. PMC13239468 42256731

[80] E. J. Ciampa , P. Flahardy , H. Srinivasan , et al., “Hypoxia‐Inducible Factor 1 Signaling Drives Placental Aging and Can Provoke Preterm Labor,” eLife 12 (2023): RP85597. 10.7554/eLife.85597 PMC10446824 37610425

[81] K.‐W. Ouyang , T.‐T. Wang , H. Wang , et al., “m6A‐Methylated Lonp1 Drives Mitochondrial Proteostasis Stress to Induce Testicular Pyroptosis Upon Environmental Cadmium Exposure,” Science of The Total Environment 931 (2024): 172938, 10.1016/j.scitotenv.2024.172938. 38703850

