# Supplementary material for: m6A‐Driven Pexophagy Triggers Placental Ferroptosis to Impair Fetal Growth Upon Environmental Stress
Source: Adv Sci (Weinh). 2026 Aug 12:e77068. Online ahead of print. doi: 10.1002/advs.77068 (PMC13464557; doi:10.1002/advs.77068)
Supplement: Supplementary file 1 [file ADVS-9999-e77068-s001.docx]

**m6A-driven pexophagy triggers placental ferroptosis to impair fetal growth upon environmental stress**

Xin-Xin Zhang ^1,2^†, Ye-Xin Luo ^1,2^†, Cheng-Fang Sun ^1,2^†, Rui Pan ^1,2^, Yu-Xi Wu ^1,2^, Zhi Yuan ^1,2^, Xin-Run Wang ^1,2^, Xu-Dong Zhang ^1,2^, Lan-Lan Wu ^1,2^, Xiang-Yu Yao ^1,2^, Zhan-Dong Song ^1,2^, Wei Chang ^1,2^, Tian Wei ^1,2^, Pan-Yuan Cai ^1,2^, Tian-Ci Zhang ^4^, Yong-Wei Xiong ^1,2^, De-Xiang Xu ^1,2^, Hua Wang ^1,2^*, Hua-Long Zhu ^1,2,3^*

**Corresponding author:** Hua Wang* [(wanghuadev@ahmu.edu.cn);](mailto:(wanghuadev@ahmu.edu.cn),) Hua-Long Zhu* (zhuhualongdev@ahmu.edu.cn)

**The Supplementary Materials file includes:**

Figures. S1-S14

Figure legends for Figs. S1-S14

Tables S1 to S6

**Fig. S1. Placental ferroptosis is positively correlated with FGR.** (A-K) A total of 183 pairs of maternal-infant samples were collected from the Fourth Affiliated Hospital of Anhui Medical University, and finally, 114 placental samples were included for a cross-sectional study. The use of all data and biological samples has obtained informed consent from the participants. (A) Workflow of the cross-sectional study. (B) Restricted cubic spline plot of MDA and birth weight. (For the three nodes, the overall P-value in the figure is less than 0.05, while the nonlinear *P*-value is greater than 0.05, indicating a nonlinear and negative correlation between MDA and birth weight.) (C) Linear regression model of MDA and birth weight. (The crude model included only two variables: MDA and birth weight. The fine model further adjusted for gestational weeks and prenatal BMI based on the crude model. Linear regression analysis revealed a significant decrease in birth weight with increasing MDA level.) (D) Restricted cubic spline plot of MDA and placental weight. (For the three nodes, the overall *P*-value in the figure is less than 0.05, while the nonlinear *P*-value is greater than 0.05, indicating a nonlinear and negative correlation between MDA and placental weight.) (E) Linear regression model of MDA and placental weight. (The crude model included only two variables: MDA and placental weight. The fine model further adjusted for gestational weeks and prenatal BMI based on the crude model. Linear regression analysis revealed a significant decrease in placental weight with increasing MDA levels.) (F) MDA levels in human placentae. (G) Linear relationship between male birth weight and MDA. (H) Linear relationship between female birth weight and MDA. (I) MDA levels in human placentae. (J and K) 4HNE immunofluorescent analysis of human placentae. *n* = 6-114. *^**^P* < 0.01 ***vs.*** AGA.

**Fig. S2. Gestational Cd exposure induces trophoblast ferroptosis and functional impairment in mouse placentae.** Pregnant mice received intraperitoneal administration of low or high doses of CdCl₂ on GD 8. Placentae were obtained on GD 16 subsequent to euthanasia. (A) Cd concentrations in maternal serum of mice. (B and C) Immunofluorescent detection of 4-HNE and Cytokeratin7 in mouse placentae. (D and E) H&E staining for mouse placentae. (F and G) Levels of TPBPA and GCM1 in mouse placentae. (H) mRNA levels of *Syna*, *Synb* and *Gcm1* in mouse placentae. (I-L) Levels of MMP2 and N-Cadherin in mouse placentae. *n* = 4-6. ^*^*P* < 0.05, ^**^*P* < 0.01 *vs.* Ctrl or 0 h.

**Fig. S3. Prenatal environmental stress induces placental ferroptosis and FGR. (**A-M) **Pregnant mice received intraperitoneal administration of low or high doses of CdCl₂ on GD 8. Placentae were obtained on GD 16 subsequent to euthanasia.** (A) Schematic diagram of fetuses and placentae. (B) Number of litters and number of pups with FGR phenotype. (C) Incidence of FGR. (D) Fetal weight. (E) Placental weight. (F) Crown-rump length of fetuses. (G) Placental diameter. (H) **Mouse placental MDA content.** (I) **Mouse placental GSH content.** (J and L) 4HNE immunostaining of placentae. (K and M) FerroOrange staining for the detection of Fe^2+^ in mouse placental primary cells. *n* = 6-8. *^**^P* < 0.01 ***vs.*** Ctrl.

**Fig. S4. Environmental stress triggers ferroptosis in human JEG-3 cells.** (A and B) Levels of GPX4 in human JEG-3 cells under different doses. (C and D) Levels of GPX4 in human JEG-3 cells at different time points. The cells were treated with CdCl_2_ (20 μM). (E-G) Levels of 4-HNE and ACSL4 in human JEG-3 cells. (H-K) Human JEG-3 cells were treated with CdCl_2_ (20 μM) for 12 h. (H and I) 4HNE immunostaining of human JEG-3 cells. (J) Quantitative image of FerroOrange in human JEG-3 cells. (K) Quantitative image of C11-BODIPY in human JEG-3 cells. *n* = 3-6. *^*^P* < 0.05, *^**^P* < 0.01 ***vs.*** 0 μM , 0 h or Ctrl.

**Fig. S5. Prenatal environmental stress induces FGR by triggering placental ferroptosis. (A**-L) On GD 8, p**regnant mice** from **the HCd and Fer‑1+HCd groups received intraperitoneal administration of CdCl₂ at 2 mg/kg.** P**regnant mice** from the Fer-1 group and Fer-1+HCd group **received intraperitoneal administration** of 2 mg/kg Fer-1 on GD 8, 9, 11, 13, and 15, with Fer-1 administered 30 min before the intraperitoneal injection of CdCl_2_. After euthanasia, the placentae were collected on GD 16. (A) Schematic diagram of fetuses and placentae. (B) **Mouse placental GSH content.** (C) **Mouse placental MDA content.** (D and E) 4HNE immunostaining of placentae. (F) TEM images of placental cells. (G and H) FerroOrange staining for the detection of Fe^2+^ in mouse placental primary cells. (I-L) Levels of GPX4, 4-HNE, and ACSL4 in mouse placentae. n = 3-6. *^**^P* < 0.01 ***vs.*** Ctrl ; *^#^P* < 0.05, *^##^P* < 0.01 ***vs.*** HCd.

**Fig. S6. Prenatal environmental stress induces accumulation of oxidized lipids in the placenta.** (A) Mouse placental oxylipidomics heatmap. (B) Names and molecular formulas of very long-chain fatty acids. (C) Immunofluorescence quantitative image of LC3B and PEX5 in mouse placental tissue. *n* = 6-7. *^**^P* < 0.01 ***vs.*** Ctrl.

**Fig. S7. *Pex5* knockdown reduces environmental stress-induced FGR and placental autophagosome count.** (A) Incidence of FGR. (B) Fetal weight. (C) Crown-rump length of fetuses. (D) Placental weight. (E) Placental diameter. (F) TEM images of mouse placental cells. *n* = 4-8. *^**^P* < 0.01 ***vs.*** Ctrl. *^##^P* < 0.01 ***vs.*** HCd.

**Fig. S8. PEX5 deficiency reverses environmental stress-induced accumulation of oxidized lipids in the placenta.** (A) C11-BODIPY immunostaining of JEG-3 cells. **The cells received CdCl₂ stimulation subsequent to *PEX5* siRNA transfection.** *n* = 6. *^**^P* < 0.01 ***vs.*** Ctrl; *^##^P* < 0.01 ***vs.*** Cd.

**Fig. S9. Prenatal environmental stress elevates lipids and H_2_O_2_ in the placenta.** (A-D) **Pregnant mice received intraperitoneal administration of low or high doses of CdCl₂ on GD 8. Placentae were obtained on GD 16 subsequent to euthanasia.** (A) TEM images of mouse placental cells. (B and C) BODIPY immunostaining of mouse placentae. (D) H_2_O_2_ levels in placentae. (E) Quantitative analysis image of BODIPY Immunostaining in the mouse placenta. *n* = 6. *^**^P* < 0.01 ***vs.*** Ctrl; *^##^P* < 0.01 ***vs.*** HCd.

**Fig. S10. Environmental stress elevates lipids and ROS in human JEG-3 cells.** (A) Levels of ACAA1, ACOX1 and CAT in JEG-3 cells. The cells were treated with CdCl_2_ (20 μM). (B-E) The human JEG-3 cells were treated with CdCl_2_ (20 μM) for 12 h. (B and C) DCFH-DA immunostaining of cells. (D and E) BODIPY immunostaining of cells. (F) Quantitative Analysis image of BODIPY Immunostaining in JEG-3 cells. *n* = 3-6. *^**^P* < 0.01 ***vs.*** 0 h, Ctrl or NC; *^##^P* < 0.01 ***vs.*** Cd.

**Fig. S11.** **Environmental stress upregulates PEX2 expression in mouse placenta and human JEG-3 cells.** (A) Levels of PEX2 in mouse placentae. **Pregnant mice received intraperitoneal administration of low or high doses of CdCl₂ on GD 8. Placentae were obtained on GD 16 subsequent to euthanasia.** (B) Levels of PEX2 in JEG-3 cells. The cells were treated with CdCl_2_ (20 μM). *n* = 3-4. *^*^P* < 0.05, *^**^P* < 0.01 ***vs.*** Ctrl or 0 h.

**Fig. S12.** **Prenatal environmental stress promotes placental m6A modification.** (A-D) **Pregnant mice received intraperitoneal administration of low or high doses of CdCl₂ on GD 8. Placentae were obtained on GD 16 subsequent to euthanasia.** (A) m6A level in mouse placentae *PEX2* mRNA. (B-D) Levels of FTO and ALKBH5 in mouse placentae. *n* = 4. *^**^P* < 0.01 ***vs.*** Ctrl.

**Fig. S13.** **Deficiency of METTL14 or ELAVL1 reverses pexophagy and placental ferroptosis upon environmental stress.** (A-B) **The cells received CdCl₂ stimulation subsequent to *METTL14* siRNA transfection.** (A) Levels of METTL14 and PMP70 in JEG-3 cells. (B) Levels of GPX4, 4HNE, and ACSL4 in JEG-3 cells. (C-D) **The cells received CdCl₂ stimulation subsequent to *ELAVL1* siRNA transfection.** (C) Levels of ELAVL1 and PMP70 in JEG-3 cells. (D) Levels of GPX4,4HNE and ACSL4 in JEG-3 cells. *n* = 3. *^*^P* < 0.05, *^**^P* < 0.01 ***vs.*** Ctrl. *^#^P* < 0.05, *^##^P* < 0.01 ***vs.*** Cd.

**Fig. S14. Prenatal SAH supplementation reverses environmental stress-induced accumulation of lipid droplets and autophagosomes in the placenta.** (A-B) On GD 8**, pregnant mice from the HCd and SAH+HCd groups received intraperitoneal administration of CdCl₂ at 2 mg/kg.** P**regnant mice from t**he SAH group and SAH+HCd group **underwent** intraperitoneal injections of 10 mg/kg SAH on GD 8, 9, 11, 13, and 15. After euthanasia, the placentae were harvested on GD 16. (A) Quantitative Analysis image of BODIPY Immunostaining in JEG-3 cells. (B) TEM images of placental cells. *n* = 6. *^**^P* < 0.01 ***vs.*** Ctrl. *^##^P* < 0.01 ***vs.*** HCd.

**Figure S15. Gestational heat exposure induces placental ferroptosis and pexophagy.** (A-E) From GD 8 to GD 18, pregnant mice were subjected to heat exposure at either low-temperature heat stress (35°C, L-HT) or high-temperature heat stress (38°C, H-HT) in an incubator, both of which are above the thermoneutral zone for mice. On GD18, the mice were euthanized, and the placentae were collected. Levels of GPX4, PEX2 , PEX5 and METTL14 in placentae. *n* = 4. ^*^*P* < 0.05, ^**^***P* < 0.01 *vs.* Ctrl.**

**Materials and methods**

**Table S1** Source and Cat No. of the primary antibody

| **Antibodies** | **Source** | **Cat No.** |
| --- | --- | --- |
| ABCD2 | Proteintech | 18179-1-AP |
| ACAA1 | Proteintech | 12319-2-AP |
| ACOX1 | Proteintech | 10957-1-AP |
| ACSL4 | Proteintech | 10957-1-AP |
| ALKBH5 | Abcam | ab195377 |
| CAT | Proteintech | 21260-1-AP |
| ELAVL1 | Abcam | ab200342 |
| FTO | Abcam | ab92821 |
| GCM1 | Proteintech | 21724-1-AP |
| GPX4 | Abcam | ab125066 |
| NBR1 | Proteintech | 16004-1-AP |
| LC3B | CST | 83506 |
| METTL14 | CST | 51104S |
| MMP2 | Proteintech | 10373-2-AP |
| N-Cadherin | CST | 13116S |
| PEX2 | Proteintech | 22613-1-AP |
| PEX5 | Proteintech | 12545-1-AP |
| PGC1α | Abcam | ab188102 |
| PMP70 | Abcam | ab109448 |
| TPBPA | Biorbyt | orb186386 |
| 4HNE | Thermo Fisher Scientific | AB_2735095 |
| β-Actin | CST | 4970S |

**Table S2** Reagent or resource.

| **Reagents** | **Source** | **Cat No.** |
| --- | --- | --- |
| Cadmium chloride | Sigma-Aldrich | 202908 |
| Ferrostatin-1 | MCE | HY-100579 |
| S-Adenosylhomocysteine | MCE | HY-19528 |
| Antibody against m6A | Synaptic Systems GmbH | 202003 |
| Actinomycin D | GLPBIO | GC16866 |
| Chloroquine | MCE | HY-17589A |
| Lipofectamine 3000 Reagent Transfection | Invitrogen | L3000-015 |
| Opti-MEM | Gibco | 34985062 |
| Hydrogen Peroxide Assay Kit | Beyotime | S0038 |
| GSH and GSSG Assay Kit | Beyotime | S0053 |
| Lipid Peroxidation MDA Assay Kit | Beyotime | S0131S |
| MEM/EBSS | Cytiva | SH30024.01 |
| Sodium Pyruvate | Solarbio | SP0100 |
| Nonessential Amino Acid Solution | Solarbio | N1250 |
| Penicillin-Streptomycin Solution | Beyotime | C0222 |
| Fetal Bovine Serum | Every Green | 11011-8611 |
| Cefazolin | MCE | HY-B1892 |
| Meloxicam | MCE | HY-B0261 |

**Table S3** Primers for real-time RT-PCR or PCR.

| **Genes** | **Sequences** | **Species** |
| --- | --- | --- |
| Gcm1 | F: AGAGCCTGTGTTGAGCAGAC  R: CTCCTGGAACCAGTCAGTCG | Mouse |
| Pex2 | F: ATGTCCACAGGATCCATGCC  R: TCTCCTCAGATGCTGTCTGT | Mouse |
| Pex5 | F: CTGGTGGAGGGCGAATGTG  R: GTCCTGGGTGAAATGGGTGG | Mouse |
| Pex10 | F: TAGCAGGGATCACGTATCTCC  R: GCACCAGATGCAAGAGGGA | Mouse |
| Pex12 | F: GCTGAGTATGGGGCTCACATC  R: CCTTGACCACATGCTGAAGAG | Mouse |
| Pex1 | F: AAGGAAGAGCGTATTAAGCTGGA  R: TCGATTTCCGCACTCTGTTCT | Mouse |
| Pex6 | F: CAGGTGGCGGGAGATGTTTTT  R: CAGCCAGGTATAAAGAGGTGTG | Mouse |
| Pex26 | F: TCCATGCCGCACTAGAGACTT  R: GAACCTGGTAGTACCGGAGGA | Mouse |
| PEX2 | F: GGAGCAGCTAGTTTGGTCCC  R: TGGCTCAAAGCGAGCTAACAG | Human |
| Syna | F: GCCCCTGGTAAACCTCACTC  R: CAAGTCCTTAGTGGGGCTGG | Mouse |
| Synb | F: ACAAAGACCTTCAAGCTGCCT  R: GCTATCTGGCCTGTGCAGTA | Mouse |
| 18S | F: GTAACCCGTTGAACCCCATT  R: CCATCCAATCGGTAGTAGCG | Mouse |
| 18S | F: CGGCTACCACATCCAAGGAA  R: GCTGGAATTACCGCGGCT | Human |

**Table S4** The sequences of the siRNAs.

| siRNAs | **Sequences** | **Species** |
| --- | --- | --- |
| *PEX2* siRNA | F: GAAGUUGGCUUUGAAUACATT  R: UGUAUUCAAAGCCAACUUCTT | Human |
| *PEX5* siRNA | F: CUGGCUUUCUGACUAUGAUTT  R: AUCAUAGUCAGAAAGCCAGTT | Human |
| *METTL14* siRNA | F: GGACUUGGGAUGAUAUUAUTT  R: AUAAUAUCAUCCCAAGUCCTT | Human |
| *ELAVL1* siRNA | F: GACCAUGACAAACUAUGAATT  R: UUCAUAGUUUGUCAUGGUCTT | Human |

**Table S5** Primers for m6A modification sites.

| **Genes** | **Sequences** | **Species** |
| --- | --- | --- |
| *Site-215* | F: CCAAAAATGCCACTGTGGGG  R: ATGGTGCACACAGCATACCA | Mouse |
| *Site-478* | F: TGTTAGCTCGCTTTGAGCCA  R: TGCACACAGCATACCACAGT | Mouse |
| *Site-898* | F: AAGCCAGGACTGTTAGCTCG  R: GTGCACACAGCATACCACAG | Mouse |
| *Site-1021* | F: ACTGTGGGGCAGTCAGTTTT  R: AACCACTTTCCCCCAATGGT | Mouse |

**Table S6.** Human clinical information.

| **No.** | **Age** | **BMI** | **Group** | **Gender** | **MDA** | **BW** | **GW** | **Smoking** | **GH** | **GDM** | **NNJ** | **NP** |
| --- | --- | --- | --- | --- | --- | --- | --- | --- | --- | --- | --- | --- |
| 1 | 26 | 18.13 | SGA1 | female | 0.91 | 2875 | 40 | No | No | No | No | No |
| 2 | 26 | 18.40 | AGA1-1 | female | 0.72 | 3310 | 40 | No | No | No | No | No |
| 3 | 26 | 18.73 | AGA1-2 | female | 0.86 | 3135 | 40 | No | No | No | No | No |
| 4 | 25 | 18.26 | SGA2 | male | 0.88 | 2845 | 40 | No | No | No | No | No |
| 5 | 29 | 18.11 | AGA2-1 | male | 0.32 | 3515 | 40 | No | No | No | No | No |
| 6 | 26 | 18.95 | AGA2-2 | male | 0.84 | 3350 | 40 | No | No | No | No | No |
| 7 | 30 | 17.19 | SGA 3 | female | 0.88 | 2935 | 40 | No | No | No | No | No |
| 8 | 29 | 19.00 | AGA3-1 | female | 0.47 | 3405 | 40 | No | No | No | No | No |
| 9 | 30 | 19.72 | AGA3-2 | female | 0.49 | 3775 | 40 | No | No | No | No | No |
| 10 | 31 | 22.43 | SGA4 | male | 1.14 | 2755 | 39 | No | No | No | No | No |
| 11 | 30 | 23.44 | AGA4-1 | male | 0.43 | 3570 | 39 | No | No | No | No | No |
| 12 | 28 | 22.10 | AGA4-2 | male | 0.71 | 4565 | 39 | No | No | No | No | No |
| 13 | 33 | 19.53 | SGA5 | male | 1.07 | 2325 | 39 | No | No | No | No | No |
| 14 | 32 | 22.94 | AGA5-1 | male | 0.54 | 3535 | 39 | No | No | No | No | No |
| 15 | 31 | 21.21 | AGA5-2 | male | 0.69 | 3200 | 39 | No | No | No | No | No |
| 16 | 24 | 20.20 | SGA6 | male | 1.18 | 2490 | 39 | No | No | No | No | No |
| 17 | 26 | 18.97 | AGA6-1 | male | 0.61 | 3405 | 39 | No | No | No | No | No |
| 18 | 27 | 18.44 | AGA6-2 | male | 0.47 | 3170 | 39 | No | No | No | No | No |
| 19 | 26 | 21.26 | SGA7 | female | 1.25 | 2290 | 38 | No | No | No | No | No |
| 20 | 29 | 20.76 | AGA7-1 | female | 0.46 | 3295 | 38 | No | No | No | No | No |
| 21 | 26 | 22.25 | AGA7-2 | female | 0.45 | 3000 | 38 | No | No | No | No | No |
| 22 | 30 | 30.04 | SGA8 | female | 1.25 | 2355 | 38 | No | No | No | No | No |
| 23 | 30 | 30.47 | AGA8-1 | female | 0.36 | 4100 | 38 | No | No | No | No | No |
| 24 | 26 | 27.34 | AGA8-2 | male | 0.64 | 3960 | 38 | No | No | No | No | No |
| 25 | 34 | 24.03 | SGA9 | female | 0.91 | 2590 | 38 | No | No | No | No | No |
| 26 | 34 | 22.86 | AGA9-1 | female | 0.55 | 3200 | 38 | No | No | No | No | No |
| 27 | 29 | 23.23 | AGA9-2 | female | 0.43 | 3145 | 38 | No | No | No | No | No |
| 28 | 39 | 30.48 | SGA 10 | female | 1.36 | 2145 | 35 | No | No | No | No | No |
| 29 | 39 | 29.32 | AGA10-1 | female | 0.46 | 3700 | 38 | No | No | No | No | No |
| 30 | 37 | 31.22 | AGA10-2 | male | 0.32 | 3600 | 38 | No | No | No | No | No |
| 31 | 38 | 24.97 | SGA11 | male | 0.99 | 1270 | 34 | No | No | No | No | No |
| 32 | 35 | 22.86 | AGA11-1 | male | 0.82 | 2845 | 36 | No | No | No | No | No |
| 33 | 37 | 22.58 | AGA11-2 | female | 1.05 | 2870 | 36 | No | No | No | No | No |
| 34 | 29 | 21.48 | SGA12 | female | 1.32 | 1800 | 34 | No | No | No | No | No |
| 35 | 30 | 20.67 | AGA12-1 | female | 1.78 | 2305 | 34 | No | No | No | No | No |
| 36 | 33 | 22.67 | AGA12-2 | male | 1.11 | 2760 | 34 | No | No | No | No | No |
| 37 | 28 | 23.88 | AGA | male | 0.57 | 3925 | 40 | No | No | No | No | No |
| 38 | 26 | 26.27 | AGA | male | 0.30 | 3950 | 38 | No | No | No | No | No |
| 39 | 34 | 32.05 | AGA | male | 0.38 | 3365 | 40 | No | No | No | No | No |
| 40 | 29 | 17.97 | AGA | male | 0.32 | 3450 | 40 | No | No | No | No | No |
| 41 | 27 | 17.97 | AGA | female | 0.38 | 3765 | 39 | No | No | No | No | No |
| 42 | 25 | 23.44 | AGA | female | 0.76 | 3830 | 40 | No | No | No | No | No |
| 43 | 29 | 17.16 | AGA | male | 0.84 | 3120 | 40 | No | No | No | No | No |

**Table S6.** Human clinical information (Continued).

| **No.** | **Age** | **BMI** | **Group** | **Gender** | **MDA** | **BW** | **GW** | **Smoking** | **GH** | **GDM** | **NNJ** | **NP** |
| --- | --- | --- | --- | --- | --- | --- | --- | --- | --- | --- | --- | --- |
| 44 | 28 | 25.09 | AGA | female | 0.75 | 3335 | 38 | No | No | No | No | No |
| 45 | 26 | 22.59 | AGA | male | 0.89 | 3870 | 40 | No | No | No | No | No |
| 46 | 36 | 22.66 | AGA | male | 0.77 | 3075 | 38 | No | No | No | No | No |
| 47 | 34 | 21.83 | AGA | female | 0.78 | 3390 | 37 | No | No | No | No | No |
| 48 | 25 | 19.33 | AGA | female | 0.76 | 3425 | 38 | No | No | No | No | No |
| 49 | 29 | 23.88 | AGA | male | 0.47 | 3690 | 39 | No | No | No | No | No |
| 50 | 29 | 21.01 | AGA | female | 1.00 | 2910 | 39 | No | No | No | No | No |
| 51 | 23 | 30.48 | AGA | male | 0.81 | 2855 | 38 | No | No | No | No | No |
| 52 | 33 | 24.22 | AGA | male | 0.60 | 3400 | 39 | No | No | No | No | No |
| 53 | 27 | 20.70 | AGA | female | 0.71 | 3310 | 40 | No | No | No | No | No |
| 54 | 26 | 24.17 | AGA | female | 0.57 | 3955 | 40 | No | No | No | No | No |
| 55 | 30 | 20.67 | AGA | male | 0.69 | 3545 | 41 | No | No | No | No | No |
| 56 | 27 | 29.29 | AGA | male | 0.63 | 3325 | 37 | No | No | No | No | No |
| 57 | 30 | 19.57 | AGA | female | 0.75 | 3670 | 39 | No | No | No | No | No |
| 58 | 30 | 20.70 | AGA | female | 0.76 | 3600 | 38 | No | No | No | No | No |
| 59 | 34 | 18.60 | AGA | female | 0.78 | 3175 | 40 | No | No | No | No | No |
| 60 | 27 | 20.00 | AGA | female | 0.58 | 3335 | 40 | No | No | No | No | No |
| 61 | 30 | 19.62 | AGA | male | 0.57 | 3065 | 38 | No | No | No | No | No |
| 62 | 33 | 21.48 | AGA | female | 0.52 | 3525 | 40 | No | No | No | No | No |
| 63 | 39 | 24.78 | AGA | female | 0.58 | 3830 | 39 | No | No | No | No | No |
| 64 | 28 | 20.20 | AGA | male | 0.63 | 4065 | 41 | No | No | No | No | No |
| 65 | 35 | 20.70 | AGA | female | 0.59 | 3270 | 39 | No | No | No | No | No |
| 66 | 27 | 19.13 | AGA | male | 0.58 | 3930 | 40 | No | No | No | No | No |
| 67 | 31 | 19.78 | AGA | female | 1.31 | 2950 | 38 | No | No | No | No | No |
| 68 | 35 | 29.67 | AGA | female | 0.73 | 3080 | 39 | No | No | No | No | No |
| 69 | 38 | 17.30 | AGA | female | 1.21 | 2805 | 38 | No | No | No | No | No |
| 70 | 28 | 18.75 | AGA | female | 0.83 | 3000 | 39 | No | No | No | No | No |
| 71 | 29 | 17.47 | AGA | male | 1.12 | 2955 | 38 | No | No | No | No | No |
| 72 | 25 | 20.31 | AGA | male | 0.73 | 3270 | 40 | No | No | No | No | No |
| 73 | 27 | 17.58 | AGA | male | 0.55 | 3695 | 40 | No | No | No | No | No |
| 74 | 32 | 23.03 | AGA | female | 0.83 | 3460 | 40 | No | No | No | No | No |
| 75 | 32 | 22.59 | AGA | male | 0.39 | 4085 | 40 | No | No | No | No | No |
| 76 | 24 | 22.04 | AGA | female | 0.45 | 3415 | 39 | No | No | No | No | No |
| 77 | 26 | 23.73 | AGA | male | 0.47 | 3155 | 38 | No | No | No | No | No |
| 78 | 27 | 22.10 | AGA | male | 0.46 | 3710 | 40 | No | No | No | No | No |
| 79 | 26 | 28.30 | AGA | male | 0.68 | 3370 | 38 | No | No | No | No | No |
| 80 | 35 | 26.67 | AGA | female | 0.42 | 4180 | 39 | No | No | No | No | No |
| 81 | 36 | 23.03 | AGA | female | 0.59 | 3985 | 40 | No | No | No | No | No |
| 82 | 31 | 22.03 | AGA | female | 0.60 | 3500 | 39 | No | No | No | No | No |
| 83 | 40 | 21.67 | AGA | female | 0.35 | 4625 | 39 | No | No | No | No | No |
| 84 | 30 | 23.81 | AGA | male | 0.66 | 3390 | 39 | No | No | No | No | No |
| 85 | 25 | 26.89 | AGA | female | 0.38 | 4085 | 39 | No | No | No | No | No |
| 86 | 33 | 20.96 | AGA | male | 0.40 | 3550 | 38 | No | No | No | No | No |

**Table S6.** Human clinical information (Continued).

| **No.** | **Age** | **BMI** | **Group** | **Gender** | **MDA** | **BW** | **GW** | **Smoking** | **GH** | **GDM** | **NNJ** | **NP** |
| --- | --- | --- | --- | --- | --- | --- | --- | --- | --- | --- | --- | --- |
| 87 | 36 | 28.40 | AGA | male | 0.44 | 4100 | 40 | No | No | No | No | No |
| 88 | 25 | 25.35 | AGA | female | 0.45 | 3800 | 39 | No | No | No | No | No |
| 89 | 30 | 20.58 | AGA | male | 0.52 | 3920 | 40 | No | No | No | No | No |
| 90 | 33 | 16.60 | AGA | male | 0.48 | 3825 | 39 | No | No | No | No | No |
| 91 | 29 | 20.96 | AGA | male | 0.51 | 3430 | 40 | No | No | No | No | No |
| 92 | 38 | 20.96 | AGA | female | 0.44 | 3170 | 39 | No | No | No | No | No |
| 93 | 31 | 22.86 | AGA | female | 0.45 | 3655 | 40 | No | No | No | No | No |
| 94 | 31 | 22.32 | AGA | female | 0.40 | 3835 | 41 | No | No | No | No | No |
| 95 | 33 | 21.67 | AGA | male | 0.59 | 3420 | 40 | No | No | No | No | No |
| 96 | 27 | 20.31 | AGA | male | 0.44 | 3925 | 38 | No | No | No | No | No |
| 97 | 40 | 20.81 | AGA | male | 0.57 | 3500 | 38 | No | No | No | No | No |
| 98 | 27 | 20.20 | AGA | female | 0.63 | 3400 | 40 | No | No | No | No | No |
| 99 | 32 | 21.48 | AGA | female | 0.45 | 3620 | 39 | No | No | No | No | No |
| 100 | 27 | 19.95 | AGA | male | 0.77 | 3140 | 38 | No | No | No | No | No |
| 101 | 30 | 19.23 | AGA | male | 0.32 | 4065 | 39 | No | No | No | No | No |
| 102 | 25 | 23.51 | AGA | male | 0.56 | 3490 | 38 | No | No | No | No | No |
| 103 | 34 | 17.42 | AGA | male | 0.56 | 3285 | 39 | No | No | No | No | No |
| 104 | 29 | 21.03 | AGA | female | 0.42 | 3245 | 40 | No | No | No | No | No |
| 105 | 22 | 21.77 | AGA | male | 0.53 | 3325 | 41 | No | No | No | No | No |
| 106 | 27 | 21.63 | AGA | male | 0.50 | 4170 | 38 | No | No | No | No | No |
| 107 | 34 | 23.63 | AGA | female | 0.60 | 3340 | 40 | No | No | No | No | No |
| 108 | 30 | 27.39 | AGA | male | 0.39 | 3940 | 40 | No | No | No | No | No |
| 109 | 24 | 26.35 | AGA | male | 0.31 | 4010 | 39 | No | No | No | No | No |
| 110 | 27 | 24.46 | AGA | female | 0.91 | 2920 | 39 | No | No | No | No | No |
| 111 | 26 | 19.46 | AGA | female | 0.34 | 3890 | 39 | No | No | No | No | No |
| 112 | 32 | 19.53 | AGA | male | 0.29 | 3600 | 39 | No | No | No | No | No |
| 113 | 31 | 23.83 | AGA | female | 0.46 | 3350 | 39 | No | No | No | No | No |
| 114 | 30 | 22.30 | AGA | female | 0.41 | 3030 | 39 | No | No | No | No | No |

**Note:** BMI: Body Mass Index (kg/m²). MDA: Malondialdehyde. BW: Birth weight (g). GW: Gestational Week. GH: Gestational Hypertension. GDM: Gestational Diabetes Mellitus. NNJ: Neonatal Jaundice. NP: Neonatal Pneumonia.
